# Supplementary material for: What is the scope of teaching and training of undergraduate students and trainees in point of care testing in United Kingdom universities and hospital laboratories?
Source: PLoS One. 2022 Aug 1;17(8):e0268506. doi: 10.1371/journal.pone.0268506 (PMC9342762; doi:10.1371/journal.pone.0268506)
Supplement: S1 Appendix — (DOCX) [file pone.0268506.s001.docx]

Appendix 1 Examples definitions of a learning outcome

| **Definition** | **Source** |
| --- | --- |
| *Learning outcomes are statements of what is expected that the student will be able to do as a result of learning the activity.* | Jenkins and Unwin, 2001 (15) |
| *Learning outcome: a statement of what a learner is expected to know, understand and/or be able to demonstrate at the end of a period of learning”.* | Gosling and Moon, 2001 (14) |
| *A learning outcome is a written statement of what the successful student/learner is expected to be able to do at the end of the module/course unit or qualification.* | Adam, 2004 (16) |
| *A learning outcome is a statement of what a learner is expected to know, understand and be able to do at the end of a period of learning and of how that learning is to be demonstrated”.* | Moon, 2002 (17) |
| *In outcome-based education, the outcomes agreed for in the curriculum guide what is taught and what is assessed.* | Harden, Crosby & Davis 1999 (18) |
| *Learning outcomes are statements of what a learner is expected to know, understand and/or be able to demonstrate after completion of a process of learning* | Kennedy, Hyland & Davis 2007 (19) |
