# Supplementary material for: What is the scope of teaching and training of undergraduate students and trainees in point of care testing in United Kingdom universities and hospital laboratories?
Source: PLoS One. 2022 Aug 1;17(8):e0268506. doi: 10.1371/journal.pone.0268506 (PMC9342762; doi:10.1371/journal.pone.0268506)
Supplement: S2 Appendix — (DOCX) [file pone.0268506.s002.docx]

Appendix 2 Suggested structures of course learning outcomes.

| Structure | Reference | Good LO example |
| --- | --- | --- |
| Begin with a consistent short stem: such as “Students will be able to” or “At course completion students “  State the desired student performance/behavior using concrete action, or operational, verbs such as create, apply, interpret, describe, identify, categorize | Schoepp 2017 (21) | Students can critically review the methodology of a research study published in a marketing journal. |
| SMART objectives  **S**pecific: What action will be performed and by whom? **M**easurable: How will success be measured? Objectives should quantify the amount of change expected.  **A**chievable: Can this objective be achieved within a given time frame and with available resources? **R**elevant: Are the objectives aligned with the instructional method and assessment?  **T**ime-Bound: When will this objective be achieved? Objectives should provide a time frame indicating when the objective will be met. | Chatterjee and Corral 2017 (22) | Upon completion of the difficult airway workshop, participants should be able to formulate an accurate algorithm for the management of an obese adult patient with inadequate face mask ventilation, according to the American Society of Anesthesiologists difficult airway algorithm |
| The ABCD method  A = Audience is the target group of the LO. The learning outcomes statement would begin something like: “The students will . . .” or “The participants will . . .”  B = Behavior The behavior is the action that will be performed by the “audience.” The action word is the “verb” in the sentence.  C = Condition A condition is the circumstance under which the behavior will take place examples include “by the end of the second session or by the start of the spring semester.”  D = Degree is the element of the learning outcome that is measurable. It basically describes how well a behavior must be accomplished. These include (“85% correct” or “with fewer than three errors”). | Carr and Hardin 2010 (23) | In response to four scenarios, each student staff member will correctly articulate the steps of the Emergency Action Plan procedures as outlined in the student staff manual. |
| The ABC method  A = antecedent or the learning activity;  B = behavior or the skill or knowledge being demonstrated  C = the criterion or the degree of acceptable performance. | Whitmann-Price and Fasolka 2010 (24) | (A) By the end of this session the learner will be able to: (B) Demonstrate sterile Foley catheter insertion (C) 100% of the time in clinical |
| Begin each learning outcome with an active verb, followed by the object of the verb followed by a phrase that gives the context. | Kennedy et al 2007 (19) | Classify reactions as exothermic and endothermic. |
