# Supplementary material for: What is the scope of teaching and training of undergraduate students and trainees in point of care testing in United Kingdom universities and hospital laboratories?
Source: PLoS One. 2022 Aug 1;17(8):e0268506. doi: 10.1371/journal.pone.0268506 (PMC9342762; doi:10.1371/journal.pone.0268506)
Supplement: S3 Appendix — (DOCX) [file pone.0268506.s003.docx]

Appendix 3 Count of verbs used in Learning outcomes

| **Row Labels** | **Count of verbs** |
| --- | --- |
| explore | 1 |
| formulate | 1 |
| Highlight | 1 |
| execute | 1 |
| undertake | 1 |
| appraise | 1 |
| benchmark | 1 |
| outline | 1 |
| conduct | 1 |
| plan | 1 |
| contribute | 1 |
| provide | 1 |
| criticise | 1 |
| receive | 1 |
| design | 1 |
| record | 1 |
| arrange | 1 |
| relate | 1 |
| consider | 1 |
| review | 1 |
| decide | 1 |
| satisfy | 1 |
| compare | 1 |
| select | 1 |
| differentiate | 1 |
| summarise | 1 |
| cost | 1 |
| theoretically underpin | 1 |
| appreciate | 2 |
| uphold | 2 |
| reflect | 2 |
| know | 2 |
| comply | 2 |
| identify | 2 |
| respect | 2 |
| acquire | 3 |
| compose | 3 |
| justify | 3 |
| use | 3 |
| aware | 4 |
| communicate | 4 |
| work safely | 4 |
| apply | 4 |
| perform | 5 |
| develop | 6 |
| know | 7 |
| recognise | 7 |
| analyse | 8 |
| describe | 9 |
| interpret | 10 |
| discuss | 10 |
| demonstrate | 11 |
| explain | 12 |
| evaluate | 13 |
| understand | 24 |
| **Grand Total** | **192** |
