# Supplementary material for: What is the scope of teaching and training of undergraduate students and trainees in point of care testing in United Kingdom universities and hospital laboratories?
Source: PLoS One. 2022 Aug 1;17(8):e0268506. doi: 10.1371/journal.pone.0268506 (PMC9342762; doi:10.1371/journal.pone.0268506)
Supplement: S4 Appendix — (DOCX) [file pone.0268506.s004.docx]

Appendix 4 Count of action verb by level of blooms taxonomy

| **Row Labels** | **Count of Masterlist (time on source lists)** |
| --- | --- |
| **Analysis** | **12** |
| Analyse | 8 |
| Appraise | 1 |
| Compare | 1 |
| Differentiate | 1 |
| Outline | 1 |
| **Application** | **33** |
| Apply | 4 |
| Conduct | 1 |
| Contribute | 1 |
| Criticise | 1 |
| Demonstrate | 11 |
| Execute | 1 |
| Interpret | 10 |
| Provide | 1 |
| Use | 3 |
| **Avoid** | **42** |
| Appreciate | 2 |
| ARRANGE | 1 |
| Aware | 5 |
| know | 9 |
| RELATE | 1 |
| SELECT | 1 |
| Understand | 23 |
| **Comprehension** | **30** |
| Discuss | 10 |
| Explain | 12 |
| Explore | 1 |
| Perform | 5 |
| Review | 1 |
| Summarise | 1 |
| **Evaluation** | **22** |
| Develop | 6 |
| Evaluate | 13 |
| Justify | 3 |
| **knowledge** | **23** |
| Acquire | 3 |
| Describe | 9 |
| highlight | 1 |
| Identify | 2 |
| Recognise | 7 |
| Record | 1 |
| **not on list** | **14** |
| benchmark | 1 |
| Cost | 1 |
| receive | 1 |
| Respect | 2 |
| satisfy | 1 |
| theoretically underpin | 1 |
| undertake | 1 |
| uphold | 2 |
| work safely | 4 |
| **Synthesis** | **15** |
| Communicate | 4 |
| Comply | 2 |
| Compose | 3 |
| Consider | 1 |
| Design | 1 |
| Formulate | 1 |
| Plan | 1 |
| Reflect | 2 |
| **SYNTHESIS 4, EVALUATION 4** | **1** |
| Decide | 1 |
| **Grand Total** | **192** |
