# Supplementary material for: What is the scope of teaching and training of undergraduate students and trainees in point of care testing in United Kingdom universities and hospital laboratories?
Source: PLoS One. 2022 Aug 1;17(8):e0268506. doi: 10.1371/journal.pone.0268506 (PMC9342762; doi:10.1371/journal.pone.0268506)
Supplement: S5 Appendix — (DOCX) [file pone.0268506.s005.docx]

Appendix 5, response to question 2 of survey 2 regarding types of evidence to meet health and care professions council standards 14.16 and 14.26

| Evidence Type | Whole laboratory response | Microbiology | Blood sciences | Haematology | Histopathology | Specialist | Immunology |
| --- | --- | --- | --- | --- | --- | --- | --- |
| Annotated work | 18 | 11.0 | 2 | 6 | 6 | 0 | 2 |
| Audit | 13 | 3.0 | 2 | 2 | 5 | 0 | 1 |
| Case study | 9 | 5.0 | 2 | 2 | 3 | 0 | 0 |
| Competency assessment | 55 | 10.0 | 9 | 8 | 12 | 1 | 1 |
| Essay/review | 23 | 9.0 | 3 | 11 | 8 | 2 | 1 |
| Journal club | 0 | 1.0 | 0 | 0 | 0 | 0 | 0 |
| Lecture/tutorial | 9 | 5.0 | 1 | 3 | 1 | 1 | 0 |
| MDT | 1 | 0.0 | 0 | 0 | 0 | 0 | 0 |
| Personal statement | 5 | 0.0 | 0 | 0 | 1 | 0 | 0 |
| Practical | 12 | 4.0 | 3 | 5 | 2 | 1 | 1 |
| Questions & Answer | 35 | 13.0 | 9 | 10 | 11 | 1 | 1 |
| Reflective statement | 61 | 22.0 | 11 | 16 | 19 | 4 | 1 |
| Self directed learning | 3 | 1.0 | 0 | 1 | 0 | 0 | 0 |
| University | 3 | 0.0 | 0 | 0 | 0 | 1 | 0 |
| Witness statement | 30 | 11.0 | 6 | 6 | 11 | 1 | 2 |
