# Supplementary material for: What is the scope of teaching and training of undergraduate students and trainees in point of care testing in United Kingdom universities and hospital laboratories?
Source: PLoS One. 2022 Aug 1;17(8):e0268506. doi: 10.1371/journal.pone.0268506 (PMC9342762; doi:10.1371/journal.pone.0268506)
Supplement: S6 Appendix — (DOCX) [file pone.0268506.s006.docx]

Appendix 6, Response to question 3 of survey 2 regarding hours trained in point of care testing

| **Laboratory** | **Useable responses** | **0 hours teaching (n)** | **Unknown hours teaching (n)** | **Range of hours** | **Mean (hours)** |
| --- | --- | --- | --- | --- | --- |
| whole trust response | 81 | 21 | 13 | 0-150 | 17.6 |
| Specialist laboratories | 7 | 0 | 2 | 1-7 | 3.4 |
| Histology/cytology | 39 | 19 | 4 | 0-15 | 2.7 |
| Immunology | 7 | 4 | 2 | 0-7 | 1.4 |
| Haematology | 29 | 4 | 6 | 0-37.5 | 6.6 |
| Biochemistry | 24 | 2 | 2 | 0-450 | 76.2 |
| Blood sciences | 21 | 1 | 1 | 0-112.5 | 17 |
| Microbiology/virology | 46 | 15 | 7 | 0-15 | 2.3 |
