# Supplementary material for: What is the scope of teaching and training of undergraduate students and trainees in point of care testing in United Kingdom universities and hospital laboratories?
Source: PLoS One. 2022 Aug 1;17(8):e0268506. doi: 10.1371/journal.pone.0268506 (PMC9342762; doi:10.1371/journal.pone.0268506)
Supplement: S7 Appendix — (DOCX) [file pone.0268506.s007.docx]

Appendix 7 Response to question 4 of survey 2 regarding training methods for point of care testing in clinical laboratories.

| **Laboratory** | **Useable responses** | **No training** | **Practical training** | **lecture/ tutorial** | **shadowing/ tour** | **Self-directed** | **E-learning** | **University** |
| --- | --- | --- | --- | --- | --- | --- | --- | --- |
| Whole trust response | 77 | 12 | 42 | 44 | 21 | 3 | 4 | 5 |
| Specialist laboratories | 7 | 0 | 6 | 5 | 3 | 1 | 0 | 1 |
| Histology/ cytology | 27 | 6 | 9 | 14 | 8 | 3 | 1 | 0 |
| Immunology | 5 | 3 | 2 | 2 | 0 | 0 | 0 | 0 |
| Haematology | 27 | 4 | 12 | 16 | 6 | 2 | 1 | 0 |
| Biochemistry | 23 | 1 | 20 | 13 | 5 | 0 | 2 | 0 |
| Blood sciences | 21 | 1 | 14 | 14 | 9 | 1 | 0 | 0 |
| Microbiology/ virology | 38 | 7 | 14 | 22 | 9 | 3 | 1 | 0 |
