# Supplementary material for: What is the scope of teaching and training of undergraduate students and trainees in point of care testing in United Kingdom universities and hospital laboratories?
Source: PLoS One. 2022 Aug 1;17(8):e0268506. doi: 10.1371/journal.pone.0268506 (PMC9342762; doi:10.1371/journal.pone.0268506)
Supplement: S8 Appendix — POCT: point of care testing. (DOCX) [file pone.0268506.s008.docx]

Appendix 8, Response to question 5 of survey 2 regarding rotations into a point of care testing section/department.

| **Laboratory** | **Useable results** | **Range of hours** | **No POCT rotation** | **N/A** | **Unknown** | **Mean** |
| --- | --- | --- | --- | --- | --- | --- |
| whole trust response | 90 | 0-450 | 56 | 7 | 10 | 18.2 |
| Specialist laboratories | 12 | 0-7.5 | 7 | 4 | 0 | 0.94 |
| Histology/cytology | 38 | 0-3.75 | 28 | 7 | 0 | 0.73 |
| Immunology | 8 | 0-7.5 | 5 | 2 | 0 | 1.25 |
| Haematology | 26 | 0-30 | 22 | 0 | 0 | 2.2 |
| Biochemistry | 23 | 0-450 | 7 | 0 | 2 | 86.7 |
| Blood sciences | 16 | 0-112.5 | 6 | 0 | 3 | 17.3 |
| Microbiology/virology | 40 | 0-15 | 23 | 6 | 4 | 1.6 |
