# Supplementary material for: What is the scope of teaching and training of undergraduate students and trainees in point of care testing in United Kingdom universities and hospital laboratories?
Source: PLoS One. 2022 Aug 1;17(8):e0268506. doi: 10.1371/journal.pone.0268506 (PMC9342762; doi:10.1371/journal.pone.0268506)
Supplement: Attachment — Submitted filename: Response to reviewers.docx [file pone.0268506.s010.docx]

RE: PONE-D-21-35377

Dear Editor,

Many thanks to both you and the reviewers for taking the time to review our manuscript and providing welcome feedback and comments. See below our response to each point raised. The authors have carefully considered the comments and tried our best to address every one of them. We hope the manuscript after careful revisions meet your standards. The authors welcome further constructive comments, if any.

Minor changes:

Authors details:

1. Line 1: Philip M. Newton. Provide the full middle name

**Response: Middle name provided**

Discussion:

1.) Line 339. Provide recommendations on possible LO that could be used to standardised POCT.

**Response: Added a paragraph suggesting liaising with laboratory colleagues and referencing the HCPC and QAA in revised LO**

Major changes:

Abstract:

1.) Line 14. Write out in full United Kingdom (UK)
2.) Line 16. Lowercase **freedom of information**
3.) Line 17, add a comma after **teaching of POCT,**
4.) Line 21. Change 12 to **Twelve**
5.) Line 22. Change 66 to **Sixty-six**
6.) Line 22. 66 laboratories replied they had no POCT training.

Change to:

**Sixty-six laboratories confirmed that there was no POCT training.**

7.) Line 23. Provide a median for the university and laboratory hours. Confirm if the range of teaching hours was 0-35 or 1-35.
8.) Line 29-32. Break into two separate sentences
9.) Line 26. 31% Is not mentioned in the results. Is this the 42 out of 117? Please add percentages to Table 4 column 5.

**Response: All revised accordingly.**

Introduction:

The introduction is too long ~10 pages versus the combined methods, results, discussion ~10.

This needs to be shortened to include only relevant information.

1.) Line 35. Write out United Kingdom (UK)
2.) Line 35. Products is lower case products
3.) Line 38. near patient testing, use lower case
4.) Line 39-43. Provide examples of commonly used POCT, and comment on the impact of COVID testing.
5.) Line 45. Change strategy to strategies
6.) Line 45 at a comma after An example is,
7.) Line 52, remove italics for will
8.) Line 65.

Fig 1 The 4 routes to Health and Care Professions Council registration for biomedical scientists (adapted from information from IBMS on becoming HCPC registered (9)).

Change to:

Fig 1. The four routes to Health and Care Professions Council registration for biomedical scientists (adapted from information from IBMS on becoming HCPC registered (9)).

Write out the full name for the abbreviations COC, IBMS, HCPC, BMS in a footnote. Provide more explanation in the figure for what is meant by top up education and equivalence process.

**Response: All revised accordingly.**

2.1

1.) Line 59-74 needs to be shortened and summarised (points to include)

- Biomedical scientists are registered with the HCPC
- What defines a biomedical scientist in terms of qualification
- Briefly describe the routes to qualification described in your figure 1
- What is a COC

**Response The portion of the manuscript has been shortened to only include the suggested relevant points.**

2.) IBMS. Write out full name when abbreviating for the first time
3.) Line 61 Biomedical scientists is given an abbreviation but this is not used consistently throughout the paper, please abbreviate all subsequent biomedical scientist to BMS

**Response: All revised, as suggested, except line 65. Biomedical scientist hasn’t been abbreviated as BMS in this instance, as “BMS” is not the protected title, “Biomedical scientist” is.**

2.2

1.) Line 76-111 needs to be shortened and summarised (points to include)

- What defines an accredited biomedical degree
- The function of the QAA benchmarking for biomedical degrees
- What QAA don’t provide i.e. national curriculum, only general guidelines
- HCPC references to POCT
- Addition of POCT to the 2019 QA benchmark statement (As compared with 2015 statement)

**Response: The portion of the manuscript has been shortened to only include the suggested relevant points.**

2.) Line 95-107. Use abbreviation **NPT**

**Response: revised accordingly**

2.3:

1.) Line 113-154 requires significant shortening (points to include)

- Include: Gosling and Moon define a learning objective as….

- There is no standard definition of a LO. Place table 1 as an appendix, and make reference to it here.

- What is the purpose of a LO

- What defines a good learning objective. Place Table 2 as an appendix, and refer to it here.

- Bloom’s Taxonomy: short comment on what it is and what it is used for.

**Response: The portion of the manuscript has been shortened to only include the suggested relevant points.**

2.4:

1.) Line 156-181: needs to be shortened and summarised (points to include)

- What is a FOI request and governed by which acts

- Who can you request this from (public sector)

- How do you request (in writing)

- Higher response rates when made via a FOI request

**Response: The portion of the manuscript has been shortened to only include the suggested relevant points.**

2.) Line 156-157: Inconsistency in capitals and lower case, freedom of information act. Please correct

**Response: revised accordingly**

3.) Line 173-176 – this is describing the method of the study (remove from the introduction)

**Response: Removed**

4.) Line 82-198: needs to be shortened and summarised to include only the aims of the research

- How much (dedicated time) POCT teaching and training is there at universities and NHS laboratories
- How is POCT taught

**Response: The portion of the manuscript has been shortened to only include the suggested relevant points.**

Methods:

1.) Line 199. Change heading to Materials and Methods
2.) Line 201. Grammar. Change to:

May 2018, with a second survey sent to all the NHS trusts/Health board laboratories in the UK in

3.) Line 202-203: Table 3 gives the wording of each of the surveys sent via freedom of information requests. Change to,

The wording for each survey is provided in Table 3

4.) Table 3: Survey 1: Point 3: correct the spelling of leaning to learning
5.) Lines 204-205

Insert the table title as a label using the caption option (above the table)

Reword the title to indicate who was assessed in survey 1 (Universities) and 2 (NHS Laboratories). Change to:

Table 3. The survey questions which were used to assess teaching and training of Point of Care Testing at universities (survey 1) and NHS laboratories (survey 2).

Provide the full name and abbreviation for IBMS, HCPC in a footnote or write it out in full in the table.

6.) Reword line 206-207. The questions used in the first survey seek to gather information on how much POCT is taught and the POCT topics covered at the selected universities.

The questions used in the first survey seek to gather information on the time allocated to POCT teaching at the selected universities and the topics covered.

**Response: revised accordingly**

7.) Reword lines 209-214.

As described in the introduction, this information will cover trainees/students going through pathways 1-3 to HCPC registration. The first survey will cover pathways 1 and 2 as students /trainees going down these pathways will have completed an accredited BMS degree, whilst the second survey covers 1-3 as all students/trainees going through these pathways will have to have a clinical laboratory placement and achieve the COC.

The separate surveys were done in order to assess the different avenues of HCPC registration of trainees/ students (refer to Fig 1). The participants in survey 1 complete an accredited BMS degree (pathways 1 and 2) whilst those of survey 2 achieve a COC during a clinical laboratory placement.

**Response: Passage reworded as suggested.**

Selection process:

1.) Line 225, 227. Please clarify how the NHS acute trust/ Health board/ Blood transfusion trusts would assist in locating pathology laboratories. Are these institutions where training Biomedical scientist are registered / perform part of their training ?

**Response: Added line explaining that these locations are where pathology laboratories are located.**

2.) Reword 228-229. This resulted in 179 FOI requests. The FOI requests were sent to each university/Trust/Health board dependent on the instructions on their internet site.

This identified 179 institutions (university/ trust/ health boards) where FOI requests could be sent.

**Response: Reworded, as suggested.**

Data analysis:

1.) Line 233. For the hours tested, the data is likely to be skewed. Check if the data is normally distributed ? (use a mean) or skewed ? (use a median).
2.) Line 233-234. Place range, median, IQR within brackets.

Response rate, response turnaround time, teaching and training hours (range, median, interquartile range) were calculated.

3.) Line 235. Add (LO) after learning objective
4.) Line 239. Add et al.

Is the action verb used on the recommended/avoid list as described by Newton et al.(29)?

5.) Line 241. Please clarify in the text, did one author assess and one author review or did all review?
6.) Line 241-242. Any changes were agreed by all 3 authors. Change to:

Any changes required agreement by all three authors.

7.) Line 242-243. Rather use et. al

Supplementary data from Newton et al.(29) was used.

8.) Line 244. It is unclear how the verbs were assigned. Was it based on the level provided by the authors (29), what is meant by ‘appeared in the majority of the lists searched’. Please reword.
9.) Line 245. Change assumed to considered
10.) Line 246, 247. If using median (based on distribution of your data), change mean to median
11.) Line 249. Change Ethical to ethics.
12.) Adjust grammar 249-251

Whilst the FOI acts allow anyone to submit FOI requests, ethics approval was obtained for the use of the FOI system in order to gather research data and the subsequent analysis and publication (Swansea Medical School Research Ethics Committee (Project reference number 2018-0030)).

**Response: revised accordingly**

Results:

4.1:

1.) Line 254. Remove all.

For the first survey to the universities offering IBMS accredited undergraduate degrees, 100%

**Response: Removed**

4.2**:**

1.) Line 260-262. Line 261 remove accredited (repeated), reworded:

Fifty-two universities responded and gave results for 55 accredited degree programs. Of these, 9 (16%) described their POCT teaching as embedded within the curriculum and so teaching hours could not be quantitated.

2.) Line 264. Need to provide a median (IQR) of hours.

The amount of direct teaching, non-embedded, received by an undergraduate on POCT ranged from 1 to 35 hours with a median (IQR) of….

3.) Does the amount of teaching not range from 0 to 35 hours?
4.) Line 266-267

Write out POCT, IBMS in full, in the title. Need to stipulate whether median hours.

5.) Line 268, 269, 273. Use LO abbreviation.
6.) Line 268: Change (FOI question 3) to (survey 1, question 3)
7.) Line 270-272. Reword to:

There were 192 action verbs, of which 54 were unique (see Appendix 1).

8.) Line 273, change 13 to Thirteen
9.) Line 275, change 47 to Forty-seven

change 42 to Forty-two

10.) Line 278, change Newton, Da Silva & Peters to Newton et al.(29)
11.) Line 278-279, please clarify, the measure for the recommended list verbs is number of appearances (? Number in total or number of LO containing recommended verbs), whilst the measure for the avoid list verbs is number of LO with the verb.
12.) Table 4 is not referenced to in the text. Please add.

Provide percentage in brackets for numbers in columns 2-5

Insert a line for total

?117 correct in the table ? 42

Write out POCT in full in the caption and define LO in the caption.

Use Table caption function

13.) Line 281. Change: The fifth criteria asked, what level of blooms would the action verb be mapped to? to :

The level of blooms taxonomy was assessed for each action verb.

14.) Refer to Table 5 in the text.
15.) Line 283. Add Newton et al.

lists described by Newton et al.(29).

Change 1 to One

16.) Line 287-288.

Insert title label using table caption option

Change to:

Table 5. Action verbs mapped to levels of Bloom’s taxonomy. The verbs were assigned using the lists collated by Newton et al.(29)

**Response: revised accordingly**

4.3:

1.) Line 289 change 4.3 POCT training in NHS to

4.3 POCT training in NHS laboratories

2.) Line 290.

Change (question 3 of the FOI) to

(survey 2, question 3)

3.) Line 290. In line 256 it states that 173 laboratories responded whilst in line 290 it states 179. Please clarify.
4.) Line 292. Change remaining laboratories giving discipline specific responses (i.e., Biochemistry, Microbiology) This resulted in 254 usable data points. Table 6 summarizes the responses. For full breakdown, please see appendix to:

remaining laboratories giving discipline specific responses (i.e. biochemistry, microbiology). This resulted in 254 usable data points. Table 6 summarizes the responses with appendix 4 providing a full breakdown.

Table 6, use the caption insert option. Change title to

Table 6. Summary of hours dedicated to teaching of Point of Care Testing in the NHS.

Change to mean to 1

Write out POCT or provide abbreviation in caption.

Lower case No and Hours in the table

5.) Line 296-301

Line 299-301 should come before line 296-298

Line 296. Provide the median (IQR)

Reword to:

Of the 254 responses, 37 stated that they could not quantify the POCT training with a further 66 reporting no POCT training. In the remaining 151, a range of 0-450 hours was dedicated to POCT training. Overall, the median (IQR) hours for teaching of POCT was …. for NHS clinical laboratories. In cases where a range of hours was provided by a respondent, the maximum value was used for consistency.

6.) Line 302 to 304.

? appendix 5

Reword to:

Fig 3, shows the different training methods used in laboratory teaching of POCT, provided in response to question 4 of survey 2. For a breakdown between the whole trust responses and the different pathology disciplines see appendix 5.

7.) Line 305-306.

Reword caption:

Fig 3. The different training methods used by UK NHS laboratories for teaching and training of Point of Care Testing in 2018.

8.) Line 307-310. As you can see in fig 3, practical training and lecturebased training was the most popular training method

Reword to:

As seen in Fig 3, practical training and lecture based training were the most popular training methods

Unclear on the statistics. 225 usable responses versus the 372 total. Please clarify in your text.

9.) Line 309, change 34 to Thirty-four
10.) Table 7. Provide % for columns 3-6

Insert title label using caption function

Write out LO and POCT in full in the table

Rename caption:

Table 7. Summary of time spent on Point of Care Testing rotation in UK NHS laboratories.

Provide a median (IQR)

11.) Line 311-315

Question 5 asked if there was any rotation into the POCT section or department for the trainee/student.

Table 7 shows the range of POCT rotations received in the response. 134 laboratories responded to this question with 90 responding for the whole trust. The remaining 163 gave discipline specific information, resulting in 253 useable responses

Reword to:

Question 5 asked if there was any rotation into the POCT section or department for the trainee/student. One hundred and thirty-four laboratories responded to this question with 90 responding for the whole trust. The remaining 163 gave discipline specific information, resulting in 253 useable responses (see Table 7).

12.) Line 317-320

Of the 253 responses, 19 stated they couldn’t quantify how much time was spent on POCT rotation. 154 responses stated that no POCT rotation took place, while a further 26 responded not applicable. The range of hours on rotation was 0-450 hours. The average time spent in a POCT rotation was16.9 hours

Reword:

Of the 253 responses, 19 stated they could not quantify how much time was spent on POCT rotation. One hundred and fifty-four responses stated that no POCT rotation took place, while a further 26 responded, not applicable. The range of hours on rotation was 0-450 hours. The median (IQR) time spent in a POCT rotation was … hours.

Provide a median (IQR)

**Response: revised accordingly**

13.) What results did you obtain for question 2 of survey 2, types of evidence. Please address in your results.

**Response: Apologies, unsure how this was missed. Results for question 2 added and discussed.**

**Discussion:**

1.) Line 322. The research was also intended to determine how much time is dedicated to POCT teaching. Please add this.
2.) Line 322-325. Separate into two separate sentences (too long).
3.) Line 324 and 326 are repetitive about the change in the QAA benchmark statement. Combine these two.
4.) Line 326. Use the FOI abbreviation
5.) Line 330. Add at universities after POCT.

The first survey is useful to gauge the baseline teaching of POCT at universities.

6.) Line 331, 332, 333, 339 use LO abbreviation
7.) Line 331. I cannot find reference of the 16 generic LO in the results?

**Response: revised accordingly**

8.) Line 332-333. Reword. The rest, which includes universities that stated they did teach POCT and gave teaching hours, provided no learning outcomes.

The remainder of universities where hours were allocated to teaching of POCT, provided no LO to students.

**Response: Reworded to:**

**“The remainder of universities provided no LO to students.”**

**Not all of the remaining universities provided hours, although some of them did.**

9.) Line 333-335. Reword

From this evidence we can infer that the majority of universities that stated they do teach POCT only do so in a non-specific way.

From this evidence we can infer that the majority of universities that teach POCT do so in a non-standardised manner.

**Response: Reworded to**

**“From this evidence we can infer that the majority of universities that teach POCT do so in a non-specific manner. “**

**We kept non-specific as the LO weren’t specific to POCT and were generic in nature**

10.) Line 335-337 it is unclear what point you wish to make, please reword.

Generic LO are expected, as there will of course be learning that is transferable across all the disciplines used by pathology and perhaps reflects the fact that POCT is not specifically mentioned in the 2015, or its predecessor, QAA benchmark statement.

**Response: Removed from manuscript**

11.) Line 341. Change couldn’t to could not
12.) Line 341-342. Remove ‘and not taught specifically’

**Response: revised accordingly**

13.) Line 340-345. Provide a comment on results you obtained for median hours taught/ trained. Do you feel that it is an adequate amount of time, what would be your recommendation? How many hours does the current QAA benchmark stipulate and elaborate on the requirements for POCT teaching and training currently in 2021?

**Response: Added comment of teaching hours and suggested increase in time to reflect growing scope.**

14.) Line 346 to 348.

At this point we need to tie this in with the second survey to the NHS pathology laboratories.

The training pathway is based on trainees/students undertaking the COC to have the core educational requirements as stated by the HCPC.

Reword:

The second survey sent to the NHS pathology laboratories assessed trainees/ students undertaking the COC to obtain the core educational requirements as stated by the HCPC.

15.) Line 348.

As the evidence from the first FOI request to the universities showed that a substantial portion of the graduates will not receive much, if any, teaching on POCT then one way to counter this would be training those graduates that do become trainee biomedical scientists.

Change to : (please check if this is the point you were wanting to make)

As the evidence from the first survey sent to the universities showed that a substantial portion of the graduates will not receive much, if any, teaching on POCT, then one way to counter this would be to formalise the POCT training of graduates that become trainee biomedical scientists.

**Response: revised accordingly**

16.) Line 352-363. This is a repetition of the results section. Instead of restating the results, speak to the 41% (26+15) that have no formal POCT training. Does this meet your suggestion of training trainee biomedical scientists (line 350-351)? Tie these thoughts together

Regarding type / method of training, don’t restate results, comment that lectures and practical were the most used. Does didactic teaching work or do you recommend a combination?

Hands on experience, again summarise the main points. 79% had no hands on experience? What is the value of hands on experience?

**Response: Added comment to address points raised.**

17.) Line 364. Change & to and
18.) Line 365. Grammar

research shows that, in most cases, the amount of POCT taught at both undergraduate level and in

change to

research shows, that in most cases, the amount of POCT taught at both undergraduate level and in

**Response: revised accordingly**

19.) Line 366-367

This research also shows that in the universities that do teach POCT it is done so mostly non-specifically, with the majority of LOs provided being generic.

Change to : (please check if this is the point you were wanting to make)

This research also revealed that in universities where POCT is taught, it is largely done so in a non-standardised or measurable way.

**Response: Reworded to**

**“This research also revealed that in universities where POCT is taught, it is largely done so in a non-specific or measurable way. “**

**Universities provided LO that weren’t specific to POCT, instead provided generic LO**

20.) Line 367-369:

With the rise in POCT being supported by pathology both teaching and training of POCT will have to increase and become more specific to meet this demand.

Change to: (please check if this is the point you were wanting to make)

With the rise in POCT being supported by the pathology discipline, both teaching and training of POCT will have to be formalised, in order to equip personnel to meet this demand.

21.) Line 370

This work creates a baseline for POCT teaching and training in the UK prior to the change in QAA

Change to:

This research provides the baseline of POCT teaching and training in the UK prior to the change in QAA

22.) Line 374. Change specialist to specialists
23.) Line 377. Add the work specific after increased calls for a
24.) Line 377. Write out COVID-19. coronavirus disease of 2019
25.) Line 384. Add a comma after changes
26.) Line 385. Lowercase Pathology to pathology

**Response: revised accordingly**

27.) Please address your results of the action verbs, taxonomy. Why is it important to use these verbs in future LO.

**Response: Added comments in discussion to address action verb use.**

5.1 Limitations:

1.) Line 390. Change 8 to **Eight**
2.) Line 395: Change 2 to **Two**

**Response: revised accordingly**

Summary:

1.) Line 397. Add Heading: Conclusion
2.) Line 398. Add it after universities
3.) Line 399-400. The concept of variable training at universities has been repeated here.

The sentence starts with universities and then talks about laboratories. Please reword.

4.) Line 401. Change & to and
5.) Line 402-404.

This was done without judgment and the information identifies a need to further develop POCT teaching and training especially in light of changes in the QAA benchmark statement and increased promotion in government guidelines and reports.

Change to : (please check if this is the point you were wanting to make)

This was done objectively and identified a need to further develop POCT teaching and training, especially in light of the changes in the QAA benchmark statement and increased endorsement in government guidelines and reports.

6.) Line 405-406

Further work is needed to see how both increased use of POCT and the QAA benchmark changes affect the training and teaching of POCT to students and Trainees in the UK .

Change to

Further research is needed to see the impact of both an increased use of POCT and the QAA benchmark changes, in the training and teaching of POCT to students and trainees in the UK.

7.) Line 407. Add an Acknowledgments section

**Response: revised accordingly**

References:

1.) Line 431, 433, 438, 499, 501. Put a space after org/
2.) Line 453, Author name is in capitals, please correct
3.) Line 504, add a Heading Supporting information

**Response: revised accordingly**

General journal guidelines:

1.) Make sure the entire text is double-spaced

**All text is now double-spaced**

Once again, thank you for taking the time for reviewing our manuscript and hopefully we’ve addressed all the feedback.

Kind regards

Lee Peters
