# Supplementary material for: What is the scope of teaching and training of undergraduate students and trainees in point of care testing in United Kingdom universities and hospital laboratories?
Source: PLoS One. 2022 Aug 1;17(8):e0268506. doi: 10.1371/journal.pone.0268506 (PMC9342762; doi:10.1371/journal.pone.0268506)
Supplement: Attachment — Submitted filename: PONE-D-21-35377_R1_Reveiwer recommendations.docx [file pone.0268506.s011.docx]

Re-review changes:

| 1. Title | Write out UK in full |
| --- | --- |
| 2. Line 38 | QAA write out in full with first abbreviation |
| 3. Line 51 | Write out INR, COVID-19 in full with first abbreviation |
| 4. Line 55 | Write out NHS in full with first abbreviation |
| 5. Line 78-81 | Check that the changes read correctly |
| 6. Line 162 | Clarify: is the freedom of information act (FOIA) a repeated statement. If not please reference  The UK FOI act (2000) and The freedom of information act (FOIA) and |
| 7. Table 1 | Please check changes are correct |
| 8. Line 246-247 | Please reword to clarify if changes refers to changes in hierarchy |
| 9. Line 362-363 | Please reword as it is unclear what the 9% refers to:  It is worth noting that only 8 of the universities gave LOs describing POCT specifically, just 9% of the Los |
| 10. Line 387-388 | Please check that the wording is correct |
| 11. Line 467 | Please clarify, are your saying that many laboratories have no POCT training or practical work experience  OR  no practical POCT work experience  Please reword |
| 12. Line 579 | Please check that the changes are acceptable |
| 13. Figure 1 | Make degree (in block 3) and biomedical scientist (middle lower block) lower case. |
| 14. Figure 2 | Write out POCT and IBMS in full for the title |
| 15. Figure 3 | Write out POCT in full for the title |
