# Supplementary material for: What is the scope of teaching and training of undergraduate students and trainees in point of care testing in United Kingdom universities and hospital laboratories?
Source: PLoS One. 2022 Aug 1;17(8):e0268506. doi: 10.1371/journal.pone.0268506 (PMC9342762; doi:10.1371/journal.pone.0268506)
Supplement: Attachment — Submitted filename: PONE-D-21-35377_R1_reviewed.docx [file pone.0268506.s012.docx]

PLOS ONE

What is the scope of teaching and training of undergraduate students and trainees in Point of Care Testing in UK universities and hospital laboratories?

--Manuscript Draft--

| Manuscript Number: | PONE-D-21-35377R1 |
| --- | --- |
| Article Type: | Research Article |
| Full Title: | What is the scope of teaching and training of undergraduate students and trainees in Point of Care Testing in UK universities and hospital laboratories? |
| Short Title: | What is the scope of teaching and training of undergraduate students and trainees in Point of Care Testing |
| Corresponding Author: | LEE PETERS  Swansea Bay University Health Board swansea, Wales UNITED KINGDOM |
| Keywords: | Laboratory training; Biomedical science; Freedom of information request; POCT |
| Abstract: | Point of Care Testing (POCT) is an analytical test performed by a healthcare professional outside of a conventional laboratory. The global POCT market was valued at US$ 23.16 billion in 2016 and is forecasted to grow to US$ 36.96 billion in 2021. This upward trend for POCT has increased workload for pathology departments who manage POCT. This research aims to characterize and analyse the teaching and training of POCT at United Kingdom (UK) universities on Institute of Biomedical Science (IBMS) accredited biomedical science degrees, and at UK hospital laboratories.  A freedom of information (FOI) request was sent in 2018 to all 52 UK universities with an accredited IBMS Biomedical science degree to request information on teaching of POCT, with a 100% response rate. Further FOI requests were sent to all NHS hospital pathology departments in the UK, regarding POCT training provided to trainee Biomedical scientists, with a 97% response rate.  Twelve of the degrees contained no POCT teaching, with a further 9 having no specific POCT teaching. Sixty-six laboratories confirmed that there was no POCT training. The university teaching hours varied between 0 and 35 hours. The median time spent teaching POCT at university was 2 hours. The laboratory teaching hours varied between 0 and 450 hours The median time spent teaching POCT in hospital laboratories was 3 hours.  A content analysis of the learning outcomes provided by 29 universities showed that only 61% (84/137) were measurable and 26% (36/137) of the learning outcomes used action verbs that have previously been listed to be avoided in learning outcome writing. Only 9%(13/137) of outcomes specifically described POCT, with 8 of these being measurable.  The findings demonstrate that although this is a commonly required skill for biomedical scientists there is a clear lack of POCT teaching and training in the UK. To meet the new QAA guidelines, but most importantly to ensure the workforce is fit for the needs of the current healthcare system, the quality and quantity of POCT teaching and training needs to improve. |
| Order of Authors: | LEE PETERS |
|  | Ana Sergio da Silva |
|  | Philip Newton |
| Response to Reviewers: | Please see response to reviewers. |
| Additional Information: |  |
| Question | Response |
| Financial Disclosure  Enter a financial disclosure statement that describes the sources of funding for the | The author(s) received no specific funding for this work. |

| work included in this submission. Review the [submission guidelines](http://journals.plos.org/plosone/s/submission-guidelines#loc-financial-disclosure-statement) for detailed requirements. View published research articles from [PLOS ONE](http://journals.plos.org/plosone/) for specific examples.  This statement is required for submission and will appear in the published article if the submission is accepted. Please make sure it is accurate. |  |
| --- | --- |
| Unfunded studies  Enter: The author(s) received no specific funding for this work.  Funded studies  Enter a statement with the following details:   - Initials of the authors who received each award - Grant numbers awarded to each author - The full name of each funder - URL of each funder website - Did the sponsors or funders play any role in the study design, data collection and analysis, decision to publish, or preparation of the manuscript? - NO - Include this sentence at the end of your statement: The funders had no role in   study design, data collection and analysis, decision to publish, or preparation of the manuscript.   - YES - Specify the role(s) played. |  |
| * typeset |  |
| Competing Interests  Use the instructions below to enter a competing interest statement for this submission. On behalf of all authors, disclose any [competing interests](http://journals.plos.org/plosone/s/competing-interests) that could be perceived to bias this work—acknowledging all financial support and any other relevant financial or nonfinancial competing interests.  This statement is required for submission and will appear in the published article if the submission is accepted. Please make sure it is accurate and that any funding sources listed in your Funding Information | The authors have declared that no competing interests exist. |

| later in the submission form are also declared in your Financial Disclosure statement.  View published research articles from [PLOS ONE](http://journals.plos.org/plosone/) for specific examples. |  |
| --- | --- |
| NO authors have competing interests  Enter: The authors have declared that no competing interests exist.  Authors with competing interests  Enter competing interest details beginning with this statement:  I have read the journal's policy and the authors of this manuscript have the following competing interests: [insert competing interests here] |  |
| * typeset |  |
| Ethics Statement  Enter an ethics statement for this submission. This statement is required if the study involved:   - Human participants - Human specimens or tissue - Vertebrate animals or cephalopods - Vertebrate embryos or tissues - Field research   Write "N/A" if the submission does not require an ethics statement.  General guidance is provided below. Consult the [submission guidelines](http://journals.plos.org/plosone/s/submission-guidelines#loc-guidelines-for-specific-study-types) for detailed instructions. Make sure that all information entered here is included in the Methods section of the manuscript. | Swansea Medical School Research Ethics Committee (Project reference number 2018-0030 |

| Format for specific study types  Human Subject Research (involving human participants and/or tissue)   - Give the name of the institutional review board or ethics committee that approved the study - Include the approval number and/or a statement indicating approval of this research - Indicate the form of consent obtained (written/oral) or the reason that consent was not obtained (e.g. the data were analyzed anonymously)   Animal Research (involving vertebrate animals, embryos or tissues)   - Provide the name of the Institutional Animal Care and Use Committee (IACUC) or other relevant ethics board that reviewed the study protocol, and indicate whether they approved this research or granted a formal waiver of ethical approval - Include an approval number if one was obtained - If the study involved non-human primates, add additional details about animal welfare and steps taken to ameliorate suffering - If anesthesia, euthanasia, or any kind of animal sacrifice is part of the study, include briefly which substances and/or methods were applied   Field Research  Include the following details if this study involves the collection of plant, animal, or other materials from a natural setting:   - Field permit number - Name of the institution or relevant body that granted permission |  |
| --- | --- |
|  |  |
| Data Availability  Authors are required to make all data underlying the findings described fully available, without restriction, and from the time of publication. PLOS allows rare exceptions to address legal and ethical concerns. See the [PLOS Data Policy](http://journals.plos.org/plosone/s/data-availability) and [FAQ](http://journals.plos.org/plosone/s/data-availability#loc-faqs-for-data-policy) for detailed information. | Yes - all data are fully available without restriction |

| A Data Availability Statement describing where the data can be found is required at submission. Your answers to this question constitute the Data Availability Statement and will be published in the article, if accepted. |  |
| --- | --- |
| Important: Stating ‘data available on request from the author’ is not sufficient. If your data are only available upon request, select ‘No’ for the first question and explain your exceptional situation in the text box. |  |
| Do the authors confirm that all data underlying the findings described in their manuscript are fully available without restriction? |  |
| Describe where the data may be found in full sentences. If you are copying our sample text, replace any instances of XXX with the appropriate details.   - If the data are held or will be held in a public repository, include URLs, accession numbers or DOIs. If this information will only be available after acceptance, indicate this by ticking the box below. For example: All XXX files   are available from the XXX database (accession number(s) XXX, XXX.).   - If the data are all contained within the manuscript and/or Supporting Information files, enter the following: All relevant data are within the manuscript and its Supporting Information files. - If neither of these applies but you are able to provide details of access elsewhere, with or without limitations, please do so. For example:   Data cannot be shared publicly because of [XXX]. Data are available from the XXX Institutional Data Access / Ethics Committee (contact via XXX) for researchers who meet the criteria for access to confidential data.  The data underlying the results presented in the study are available from (include the name of the third party | All relevant data are within the manuscript |

| and contact information or URL).  • This text is appropriate if the data are owned by a third party and authors do not have permission to share the data.  * typeset |  |
| --- | --- |
| Additional data availability information: |  |

Manuscript	[Click here to access/download;Manuscript;manuscript.docx](https://www.editorialmanager.com/pone/download.aspx?id=30681557&guid=6811cf43-5043-4212-9477-f04366914827&scheme=1)

**What is the scope of teaching and training of undergraduate students and trainees in point of care testing in United Kingdom universities and hospital laboratories?**

# 1 Lee Peters*^[1]^, Ana Sergio Da Silva^1^, Philip Mark Newton^1^

2 ^1^ Research in Health Professions Education, Swansea University Medical School, Swansea 3 University, Swansea, Wales, United Kingdom SA2 8PP

4***Correspondence:**
5 Corresponding Author
6 141750@swansea.ac.uk
7 **Keywords:** Laboratory training, Biomedical science, Freedom of information request, HCPC,
8 POCT

# 9 1 Abstract

10 Point of care testing (POCT) is an analytical test performed by a healthcare professional outside
11 of a conventional laboratory. The global POCT market was valued at US$ 23.16 billion in 2016
12 and is forecasted to grow to US$ 36.96 billion in 2021. This upward trend for POCT has increased
13 workload for pathology departments who manage POCT. This research aims to characterize and 14 analyse the teaching and training of POCT at United Kingdom (UK) universities on institute of

15	biomedical science (IBMS) accredited biomedical science degrees, and at UK hospital 16	laboratories.

A freedom of information (FOI) request was sent in 2018 to all 52 UK universities with an accredited IBMS biomedical science degree to request information on teaching of POCT, with a 100% response rate. Further FOI requests were sent to all national health service hospital pathology departments in the UK, regarding POCT training provided to trainee biomedical scientists, with a 97% response

rate.

Twelve of the degrees contained no POCT teaching, with a further 9 having no specific POCT teaching. Sixty-six laboratories confirmed that there was no POCT training. The university teaching hours varied between 0 and 35 hours. The median time spent teaching POCT at university was 2 hours. The laboratory teaching hours varied between 0 and 450 hours The median time spent teaching POCT in hospital laboratories was 3 hours.

A content analysis of the learning outcomes provided by 29 universities showed that only 61% (84/137) were measurable and 26% (36/137) of the learning outcomes used action verbs that have previously been listed to be avoided in learning outcome writing. Only 9%(13/137) of outcomes specifically described POCT, with 8 of these being measurable.

The findings demonstrate that although this is a commonly required skill for biomedical scientists, there is a clear lack of POCT teaching and training in the UK. To meet the new QAA guidelines, but most importantly to ensure the workforce is fit for the needs of the current healthcare system, the quality and quantity of POCT teaching and training needs to improve.

# 2 Introduction

The United Kingdom (UK) Medicines and healthcare products regulatory agency (MHRA) define point of care testing (POCT) as any analytical test performed for a patient by a healthcare professional outside of the conventional laboratory setting (1). Other terms commonly used to describe POCT include near patient testing (NPT) and bedside testing.

POCT use is increasing, with the global POCT market expected to grow from US$ 23.16 billion in 2016 to US$ 36.96 billion in 2021 (2). This increasing demand has been attributed to increasing clinical demand, heavy industry promotion, short turnaround time for results, economical and practical factors, and advancements in technology (3). Examples of POCT include glucose meters, pregnancy tests, INR and coronavirus disease of 2019 (COVID-19). The global pandemic has exacerbated demands for POCT. To meet this growing demand, laboratory staff will need to be trained to support and deliver this service.

The growth and development of POCT has been identified in key UK government healthcare strategies. An example, is ‘Looking forward: Healthcare science in NHS Wales’ (4). This document published by the Welsh government gives POCT in the community as an example of a new role for healthcare scientists. In 2019 the Welsh government also published the pathology statement of intent (5). This document lists POCT as a pathology specialty and that it is one of the fields that is *“... rapidly evolving, driven by a reduction in costs, increased connectivity and technological innovation”.* The statement of intent also discusses how POCT will be part of the service design of pathology in Wales. The Scottish healthcare science national delivery plan 2015-2020 (6), lists POCT as one of the five service improvement programmes that will *“… deliver high-quality, sustainable health and care services for Scotland…”.* The NHS England long-term plan (7) discusses the use of POCT as a method to prevent hospital admissions. The ‘Science in Healthcare: delivering the long-term plan’ report (8) from the chief scientific officer of England, lists POCT as an emerging technology that will shape healthcare science and a key component in care

pathways.

## 2.1 Biomedical science education in the United Kingdom

The health and care professions council (HCPC) is the regulator of 15 health and care professions in the UK. The professions include physiotherapists, radiographers and biomedical scientists

(BMSs). BMS is the protected title of a laboratory scientist that has met the HCPC standards of proficiency. Figure 1 shows the routes to HCPC registration for BMSs

Figure 1. The four routes to health and care professions council registration for biomedical scientists

‘Top-up’ education: refers to supplementary education needed to meet the HCPC requirements. Equivalence process: refers to the route used for laboratory scientists that work at the level of a BMS and want to register as a BMS but that are not HCPC registered.

(adapted from information from institute of biomedical science on becoming

HCPC registered (9)).

COC: certificate of competence; IBMS: institute of biomedical science; HCPC: health and care professions council, , BMS:biomedical scientist ,

The institute of biomedical science (IBMS) certificate of competence (COC) is a qualification that demonstrates the student/trainee meets the HCPC standards of proficiency for registration as a BMS (10). In figure 1 routes one and two, students will undertake an IBMS accredited undergraduate degree and laboratory training either during or after completion of the degree. Route 3 is for students that do not have an accredited degree and require educational ‘Top-up’s’, to ensure they meet the educational requirements, and clinical laboratory training. Route 4 is an equivalence route to registration. This route was not part of this research due to low numbers and a non-standardised training program.

## 2.2 Institute of biomedical science degree accreditation and the United Kingdom Quality Assurance Agency

Undergraduate degree accreditation by the IBMS ensures the degree covers the specified subjects, in specific depth, to meet the HCPC standards of proficiency for BMS. Degrees are only accredited if they incorporate the requirements detailed in the Quality Assurance Agency for higher education (QAA) subject benchmarking for biomedical science (11). The accreditation process also looks at other aspects of the program, such as program delivery and placement opportunities (12).

The QAA is an independent body that monitors and advises on standards and quality in the UK higher education. QAA benchmark statements define the academic standards that can be expected of a graduate in terms of what they might know, do and understand at the end of their studies, and describe the nature of the subject (11). The QAA initiates regular reviews of their content, or in response to significant changes in the discipline (11). They do not provide a national curriculum for a subject but provide general guidance for writing the learning outcomes (LOs) of the course.

POCT is referenced twice in the HCPC standards of proficiency for BMS:

1. Standard 14.16, know the extent of the role and responsibility of the laboratory with respect to the quality management of hospital, primary care and community-based laboratory services for NPT and non-invasive techniques.
2. Standard 14.26, be able to use standard operating procedures for analyses including POCT in vitro diagnostic devices

Students or trainees undertaking the COC must meet all the standards of proficiency, including the standards shown in table 1. To become IBMS accredited, undergraduate courses only have to meet the QAA benchmarking statement.

The QAA benchmark statement released late 2019 (11) now includes NPT under the clinical chemistry requirements. All universities seeking IBMS accreditation or re-accreditation post 2019 will have to evidence how they meet the new benchmark statement. NPT has been added to the benchmark statement *“the principles and applications of biochemical investigations used for screening, diagnosis, treatment and monitoring of disease, including near-patient testing”.* POCT has been added *“in light of recent advances in practice”.* (11)

POCT was not specifically mentioned in the previous QAA biomedical science statement (13).

This research was conducted before the new QAA benchmark statement was released in late 2019. Therefore, universities who responded to the FOI request were accredited under the 2015 benchmark statement.

## 2.3 Learning outcomes

LOs are a statement of what a learner is expected to know and be able to demonstrate by assessment after learning. Gosling and Moon (14) state that;

*“Well written learning outcomes provide a means of mapping the content of a curriculum – for example, to see how they reflect benchmark statements, which of the key skills are acquired, where the same skill or content is appearing more than once in the programme, the capabilities the students acquire as they progress through the levels in the programme of study.”*

There appears to be no standard definition of a LO, several different definitions have been suggested, see appendix 1. Whilst there is no standard definition there is a conserved theme.

Kennedy, Hyland & Davis (19), stated that the various definitions they cited did not differ significantly and it was clear that LOs focus on what the learner has achieved and what the learner can demonstrate at the end of a learning activity.

The use of LOs has been reported to have many benefits. They enable internal and external quality assurance to determine how appropriate the curriculum is (14). The LOs allow students to gauge what they can achieve if they participate on a module/course. Mahajan and Sarjit Singh (20) listed a range of perceived benefits that in their opinion included; helping the student choose an appropriate course, give a clear idea to the students of what are they going to learn or achieve at the end of the class before the start of every class, help teachers design their teaching material more effectively, help teachers select appropriate strategies for teaching and make assessments mapping clear and easy. Given the importance of LOs throughout the years many theorists have suggested approaches to their design. Some suggested structures for a good LO are described in appendix 2

LOs use verbs to describe the expectations of the student. Bloom's Taxonomy, first published in 1956 (26) and revised in 2002 (27), is often used to write LOs. It classifies thinking skills into six hierarchically organized categories that range from lower-level (know and understand) to higher-order (apply, analyze, evaluate, create) (26). Each of the levels have indicative verbs. Stanny (28), and our previous work (29), analyzed examples of verb lists on educational websites in the United States of America and the UK respectively. Both studies found little consistency in the lists, including several verbs that appeared in all levels of the hierarchy suggested by Bloom. We have previously suggested a list of verbs to use at each hierarchical level and a list of verbs to avoid using in writing LOs (29).

## 2.4 Freedom of Information requests

The UK FOI act (2000) and The freedom of information act (FOIA) and FOI (Scotland) act (2002) (30) gives the right to access recorded information held by public sector bodies. The FOI act has some limitations about what information is covered by the act, e.g. cost above the allowable threshold, repeat of a previous request or the request is vexatious. To make a FOI request, the organisation must be contacted in writing, e.g. e-mail, letter, online form and requires your name, contact details and detailed description of the information required. The legislation allows 20 working days for a response.

FOI requests have been used as a mechanism to distribute surveys, questionnaires, etc. to gather data for research. Fowler et al (31) conducted a systematic review of the use of the UK FOI act to gather data for healthcare research. The 16 studies reviewed covered a range of requests such as litigation, surgical provision and laboratory provision. One of the studies included in the review tried to obtain data via a letter and achieved a response rate of 11%. When they made the same request via FOI the response rate rose to 83%. FOI requests have also been used in educational research. Knight, Stead & Geyton (32) used a FOI to look at the academic performance of graduate-entry and undergraduate medical students at a UK medical school.

Phillips et al (33) reported a response rate between 26.6% and 100%, with a mean of 71.3% (SD 19.5%) when they looked at survey response rates in three education journals. They compared this to other reported response rates; United states of America government surveys (~ 70%), and other surveys across Europe (50-60%). Health profession education research using FOI requests have shown a response rate of 97%, 93%, 83% (Arulrajah and Steele (34), Ayton and Ibrahim (35), Copeland and Barron (36)) respectively.

The research presented here is a pragmatic approach to answer the research questions to inform further research into the BMS curriculum and laboratory training with regards to POCT teaching and training.

The current state of POCT teaching in universities and clinical laboratories is unknown and this research seeks to answer the following research questions:

1. How much POCT is taught in UK accredited undergraduate biomedical science degrees pre change in QAA benchmark statement?
2. How POCT is taught in UK accredited undergraduate biomedical science degrees pre change in QAA benchmark statement?
3. How much POCT is taught in UK pathology laboratories pre change in QAA benchmark statement?
4. How POCT is taught in UK pathology laboratories pre change in QAA benchmark statement?

# 3 Materials and Methods

A survey was sent to all UK universities offering IBMS accredited biomedical science degrees in

May 2018, with second survey sent to all the NHS trusts/health board laboratories in the UK in October/November 2018. The Surveys were sent using a FOI request. The wording for each survey is provided in Table 1.

| **Survey 1 (n=52)** | **Survey 2 (n=179)** |
| --- | --- |

|  | For each pathology discipline in your  trust/health board (i.e. microbiology,  haematology, blood transfusion,  biochemistry, histopathology etc…); |
| --- | --- |
| 1. Please could you list all your institute of biomedical science (IBMS) accredited courses your institution currently run? | 1. On average how many trainee biomedical scientist/university biomedical science placement students do you have a year? |
| 2. Approximately how many hours of teaching time is dedicated to point of care testing/ near patient testing on each of your institute of biomedical science (IBMS) accredited courses? | 2. What types of evidence are used by your students/trainees to meet the Health and Care Professions Council (HCPC) standards of proficiency for biomedical scientists for standards 14.26 and 14.16 in the certificate of competence i.e. reflective sheet, essay, competency etc…? |
| 3. Please could you provide any learning outcomes relating to point of care testing/ near patient testing and how are they assessed? | 3. Approximately how many hours of  teaching/training is dedicated to point of care testing / near patient testing for each student/trainee? |
| 4. Please could you provide the learning outcomes used to meet the health and care professions council (HCPC) standards of | 4. What types of teaching/training do you give these students/trainees in point of care |

| proficiency for biomedical scientists for standards 14.26 and 14.16 and how are they assessed? | testing i.e. seminars, practical training etc…? |
| --- | --- |
|  | 5. Do your students/trainees get a  secondment/rotation into a point of care section and if so, for how long? |

# Table 1. The survey questions which were used to assess teaching and training of point of care testing at universities (survey 1) and national health service laboratories (survey 2)

The questions used in the first survey seek to gather information on time allocated to POCT teaching at the selected universities and the topics covered. The questions used in the second survey seek to gather information on how the pathology laboratories train their trainee BMS on POCT, including how they met the relevant HCPC standards. The separate surveys were done in order to assess the different avenues of HCPC registration of trainees/students (refer to Figure 1). The participants in survey one complete an IBMS accredited degree (Figure 1, pathways 1 and 2) whilst those of survey 2 achieve a COC during a clinical placement or training position. The second survey asked the laboratories to provide information for all relevant disciplines in their hospital trust or health board. This was to capture practices per pathology discipline, if there was no pan pathology approach to training.

The surveys were sent a few months apart due to the large number of requests being sent out and to allow time to deal with queries.

## 3.1 Selection process

A list of UK-based IBMS-accredited degrees was obtained from the IBMS website (39). Only UK universities were selected as the aim of the research was to identify POCT training in the UK. This resulted in 52 university FOI requests.

Despite an extensive search, there was no list of pathology laboratories in the UK. Pathology laboratories are the main provider of employment of BMS. Therefore, an FOI request was sent to every NHS acute trust or health board in the UK. To identify these, the websites for NHS England, Wales, Scotland and Northern Ireland were searched. Additionally, the public health and blood transfusion trusts for each nation were also added to the list. The trusts and health boards were selected as they host pathology as part of their organisation. This identified 179 institutions (trusts/health boards) where FOI requests could be sent.

The FOI requests were sent to each university/trust/health board dependent on the instructions on their internet site.

## 3.2 Data Analysis

All responses were recorded in excel for analysis. Each response was anonymised and given a unique identifier to avoid any potential reputational damage to the universities and laboratories. Response rate, response turnaround time, teaching and training hours (range, median and interquartile range) were calculated. If range of training hours was provided, then the maximum value was used.

LOs were reviewed and assessed against the following criteria:

1. Was the LO measurable?
2. Does the LO specifically describe POCT?
3. Does the LO have multiple outcomes?
4. Is the action verb used on the recommended/avoid list as described by Newton et al (29)?
5. What level of blooms taxonomy would the verb be mapped to?

The LOs were assessed by at least one author and then reviewed by the other two authors. Any changes required agreement by all three authors.

To assign the verb to a level of blooms taxonomy, the authors used the supplementary data from Newton et al (29). The supplementary data includes the verb lists from 47 UK higher education institutions. Verbs were assigned to the level where it appeared in the majority of these lists.

For training hours, a day was considered to be 7.5 hours and a week 37.5 hours unless otherwise stated. When calculating median, if the respondent gave a range of time, then the highest value was used to calculate the mean. Unknown responses were not included in the calculation.

## 3.3 Ethics

Whilst the FOI acts allow anyone to submit FOI requests, ethics approval was obtained for the use of the FOI system in order to gather research data and the subsequent analysis and publication

(Swansea Medical School Research Ethics Committee (Project reference number 2018-0030).

# 4 Results

## 4.1 Response rate & turnaround time

For the first survey to the universities offering IBMS accredited undergraduate degrees, 100% (52) responded, while for the second survey to the UK trusts/health boards with clinical laboratories, 97% (173) responded.

77% (40) of the universities responded within the 20-working day limit, whilst 84% (146) of the trusts/health boards who responded did so within the limit.

## 4.2 Point of care training in universities

Fifty-two universities responded and gave results for 55 accredited degree programs. Of these, nine (16%) described their POCT teaching as embedded within the curriculum and so the teaching hours could not be quantitated. Twelve programs (22%) stated that there were no specific teaching hours dedicated to POCT.

The amount of direct teaching, non-embedded, received by an undergraduate on POCT ranged from 0 to 35 hours with a median (IQR) of 2 hours (0.25-5.75). Figure 2 shows the range of POCT teaching hours provided by the universities in response to question 2 of survey 1.

Figure 2. Point of care testing teaching hours by number of degrees programmes on institute of

biomedical science accredited degree in the United Kingdom 2018

When asked to provide the LOs for teaching on POCT (survey 1 question 3), 29 universities (56%) responded with at least one LO. The range of LOs provided per university was 1-31 with 137 LOs provided in total. There were 192 action verbs, of which 54 were unique (see appendix 3).

The analysis found that of the 137 LOs, only 61% (84/137) were considered measurable. Thirteen of the LOs provided specifically mention POCT and of these 8 were measurable. See table 2 for summary of analysis.

Forty-seven of the provided LOs had multiple outcomes described. Forty-two of these had 2 outcomes, 1 had 3, and 4 had 4 outcomes described per LO.

We also considered whether the LO action verb appeared on the recommended/avoid list described by Newton et al (29). Verbs on the recommended list appeared in 49% (67/137) of LOs, whilst 26% (36/137) of the LOs had an action verb from the avoid list, see appendix 4.

|  | Is the LO measurable? | Does the LO describe  POCT  specifically? | Does the LO contain multiple outcomes? | Does the LO contain an action verb on the  Recommended list? |
| --- | --- | --- | --- | --- |
| Yes | 84 (61%) | 13 (9%) | 47 (34%) | 67 (49%) |
| No | 53 (39%) | 124 (91%) | 90 (66%) | 93 (68%) |

**Table 2. Analysis of the 137 point of care testing learning outcomes from universities providing institute of biomedical science accredited degrees in the United Kingdom.**

**LO: learning outcomes**

**POCT: point of care testing**

The level of blooms taxonomy was assessed for each action verb. Of the 54 unique verbs used, 37 could be assigned to a level on blooms taxonomy, 7 were on the avoid list and 9 verbs were not found on any of the lists described by Newton et al (29). One verb, decide, was unmapped as it was split between the synthesis and evaluation levels, with 4 lists placing it on the synthesis and

4 on the evaluation level, see table 3. For the full list, please see appendix 4.

| level of blooms taxonomy | Count of verbs assigned to level |
| --- | --- |
| Evaluation | 3 |
| Unmapped | 1 |
| Synthesis | 8 |
| Analysis | 5 |
| Application | 9 |
| Comprehension | 6 |
| Knowledge | 6 |
| Not on list | 9 |
| Verbs to Avoid | 7 |

# Table 3. Action verbs mapped to level of Bloom’s taxonomy. The verbs were assigned using the lists collated by Newton et al. (29)

## 4.3 Point of care training in national health service laboratories

Table 4 Shows the evidence used by clinical laboratories to meet requirements for HCPC standards of proficiency 14.16 and 14.26, provided in response to question 2 of survey 2 (Table 1). Of the 173 laboratories surveyed, 118 responded. Of these, 76 gave a response for their whole organisation, with the 42 remaining laboratories giving discipline specific responses (i.e. biochemistry, microbiology). This resulted in 181 usable responses. These laboratories used 15 different types of evidence to meet the specified standards. For a breakdown between the whole trust responses and the different pathology disciplines, see appendix 5.

| Evidence Type | Number of times used | % |
| --- | --- | --- |
| Reflective statement | 134 | 22.7 |
| Competency assessment | 96 | 16.2 |
| Questions & Answer | 80 | 13.5 |
| Witness statement | 67 | 11.3 |
| Essay/review | 57 | 9.6 |
| Annotated work | 45 | 7.6 |
| Practical | 28 | 4.7 |
|  |  | 4.4 |
| Audit | 26 |  |
| Case study | 21 | 3.6 |
| Lecture/tutorial | 20 | 3.4 |
| Personal statement | 6 | 1.0 |
| Self-directed learning | 5 | 0.8 |
| University work | 4 | 0.7 |
| Journal club | 1 | 0.2 |
|  |  | 0.2 |
| MDT (multidisciplinary **team meeting)** | 1 |  |

# Table 4. Types of evidence used by United Kingdom clinical laboratories to meet requirements for health and care professions council standards of proficiency 14.16 and 14.26

For the total number of hours spent training on POCT (table 1, survey 2, question 3), 147 out of the 173 laboratories responded. Of these, 81 gave a response for their whole organisation, with the 66 remaining laboratories giving discipline specific responses (i.e. biochemistry, microbiology). This resulted in 254 usable data points. Table 5 summarizes the responses with appendix 6 providing a full breakdown.

| Useable results | Range of hours | Responses with POCT teaching | Responses with no POCT teaching | Unknown hours POCT teaching | Median  (hours) and interquartile  range [Q1-  Q3] |
| --- | --- | --- | --- | --- | --- |
| 254 | 0- 450 | 151 | 66 | 37 | 3 [0-7.5] |

# Table 5. Summary of hours dedicated to teaching point of care testing in the national health service

# POCT: point of care testing

Of the 254 responses, 37 stated they could not quantify the POCT training, with a further 66 reporting no POCT training. In the remaining 151, a range of 0-450 hours was dedicated to POCT training. Overall, the median (IQR) hours for teaching POCT was 3 hours (0-7.50) for NHS clinical laboratories. In cases where a range of hours was provided by a respondent, the maximum value was used for consistency.

Figure 3 shows the different training methods used in laboratory teaching of POCT, in response to question 4 of survey 2 (Table 1). For a breakdown between the whole trust responses and the different pathology disciplines see appendix 7.

Figure 3. The different training methods used by United Kingdom national health service laboratories for teaching and training of point of care testing in 2018.

This question yielded 225 usable responses. Several responses listed multiple training methods. As seen in figure 3, practical training and lecture-based training was the most popular training method, with 119 and 130 responses respectively of the 372 overall responses. Thirty-four responses listed no training in POCT, with 6 citing learning at university as the method for POCT training.

Question 5 of survey 2 (Table 1) asked if there was any rotation into the POCT section or department for the trainee/student. One hundred and thirty-four laboratories responded to this question with 90 responding for the whole trust. The remaining laboratories gave discipline specific information, resulting in 253 useable responses (see table 6)

| Useable results | Range of hours | Responses with  POCT rotation | No POCT rotation | N/A | Unknown | Median  (hours) and  Interquartile range [Q1-  Q3] |
| --- | --- | --- | --- | --- | --- | --- |
| 253 | 0-450 | 54 (21%) | 154 (61%) | 26 (10%) | 19 (8%) | 0 [0-3.75] |

# Table 6. Summary of time spent on point of care testing rotation in United Kingdom laboratories

Of the 253 responses, 19 stated they could not quantify how much time was spent on POCT rotation. One hundred and fifty-four responses stated that no POCT rotation took place, while a further 26 responded, not applicable. The range of hours on rotation was 0-450 hours. The median (IQR) time spent in a POCT rotation was 0 (3.75) hours. For a full breakdown of responses, see Appendix

8.

# 5 Discussion

The purpose of this research was to characterize and analyse what, and how much POCT is taught at undergraduate level and to those undertaking the IBMS certificate of competence to enter the HCPC register as a BMS.

Both surveys, sent via a FOI request, were sent before the new QAA bench marking statement for biomedical sciences was released in late 2019, so any university seeking accreditation 2020 onwards will have to prove they meet the new QAA statement. This development is bound to affect the amount of POCT taught on these programs.

The first survey is useful to gauge the baseline teaching of POCT at universities. It is worth noting that only 8 of the universities gave LOs describing POCT specifically, just 9% of the LOs. The remaining 21 universities gave generic laboratory LOs. The remainder of universities, provided no LOs to students. From this evidence we can infer that the majority of universities that teach POCT only do so in a non-standardised manner. Our study revealed that only 61% of LOs were measurable. Schoepp, in their review of LO in 10 leading universities in 2017, found that only 54% of LO were measurable (22). The verbs were assigned to all 6 levels of the blooms taxonomy, with the LO spread throughout the levels, except evaluation. Scoepp, also mapped the LOs to all levels, however, they found that the middle order, application and analysis, dominated. (22). Thirty-one percent of the LOs used action verbs from the avoid list and 66% contained multiple outcomes, therefore not meeting the described structures for a good LO. It is therefore important that universities review their LO so that future employers will have a clear idea what their trainees/students have been taught. The addition of POCT to the requirements of degree accreditation will require universities to include more POCT specific LOs to their curriculum. We suggest that universities engage with clinical laboratories when writing POCT specific LOs to reflect current practice and refer to both the HCPC standards and QAA benchmark statement for guidance.

Twelve of the universities responded that they had no POCT teaching on their courses and a further nine could not give an exact teaching time as POCT was embedded within the curriculum. The median teaching time was 2 hours, and whilst the 2019 QAA benchmark does not stipulate teaching hours, this appears low. The increasing scope of POCT, across the range of disciplines of pathology, merits inclusion across the biomedical science degree curriculum. The LOs provided were mostly generic and were not reflective of the range of POCT equipment available.

From the university’s perspective, not all graduates from accredited biomedical science degrees become HCPC registered BMS. Therefore, as they only had to meet the 2015 QAA benchmark statement for IBMS degree accreditation, fitting in all topics relevant to BMS may not have been a priority or been able to fit into their teaching program.

The second survey sent to the NHS laboratories assessed trainees/students undertaking the COC to obtain the core educational requirements as stated by the HCPC.

The training pathway is based on trainees/students undertaking the COC to have the core educational requirements as stated by the HCPC. As the evidence from the first survey sent to the universities showed that a substantial portion of the graduates will not receive much, if any teaching on POCT, then one way to counter this would be to formalize the POCT training of graduates that do become trainee BMS.

The second survey to the NHS clinical laboratories asked the question of how many hours are dedicated to POCT teaching. Forty-one percent of the returns stated that they either had no POCT training or the POCT teaching was unknown. This is a surprising finding, as all trainee BMS require POCT teaching/training to meet the HCPC requirements for registration. This also means that due too no, or often limited teaching hours of POCT at university, a large percentage of newly qualified BMS will not have the skills or knowledge to meet the requirements for the increased use of POCT. In their national review of english pathology, Lewis et al (42), the authors looked at the percentage of hospital coagulometer, community glucose and community INR tests were done with laboratory oversight. They found that 24%, 65% and 69% respectively of these tests were outside of laboratory oversight and recommend “*All POCT, wherever it is undertaken, is performed to these agreed quality standards, and under a governance structure that is linked to and supported by an accredited lab*”

A subsequent question asked what types of POCT training was provided in the laboratory for trainees/students. The two most used training techniques were lecture based and practical demonstration, 35% and 32 respectively. It is interesting to note that the two HCPC standards relating to POCT have different outcomes. Standard 14.16 expects BMS to have knowledge of the role and responsibilities of the laboratory with respect to POCT, whilst standard 14.26 expects BMS to have a practical knowledge of POCT analysis. A combination of the two teaching methods therefore seems appropriate.

The final question of the second survey asked about secondments to a POCT section or department.

This question relates to the ‘hands-on’ POCT experience that biomedical science trainees experience. Only 21% of respondents provided the designated hours for a secondment. Therefore, 79% of trainees will have no practical experience of POCT in a clinical environment once qualified. This is backed up by the response to question 2 of survey 2 (table 1), asking for the types of evidence used to meet the two HCPC standards with POCT. In 21%, the evidence related to ‘hands on’ experience, competency assessments and practical. This further reinforces the possibility of a gap in knowledge in POCT for newly qualified BMS.

The question now becomes - does the potential gap in POCT teaching and training matter ? This research shows, that in most cases, the amount of POCT taught at both undergraduate level and in pathology laboratories is limited. This research also revealed that in the universities where POCT is taught, it is largely done so in a non-specific or measurable way. With the rise in POCT being supported by pathology, both teaching and training of POCT will have to be formalized, in order to equip personnel to meet this demand.

This work provides the baseline for POCT teaching and training in the UK prior to the change in QAA benchmark statement for biomedical science. The challenge now is for universities providing IBMS accredited degrees to provide this extra teaching. These universities already have close links with pathology laboratories as part of their teaching and consideration should be given to utilizing POCT specialists to support university teaching of POCT.

The research also highlights the lack of specific POCT teaching. The IBMS launched a qualification in 2019 in POCT, aimed at BMS working, or with an aspiration to work in POCT.

This was in response to increased calls for a specific qualification (40). The COVID-19 pandemic has put a spotlight on POCT due to the use of COVID -19 testing kits and will result in increased exposure to POCT in health systems (41). How this impacts the training and education of laboratory staff in POCT will take some time to discover.

The addition of POCT into the QAA benchmark statement and therefore to the IBMS degree accreditation process will impact universities providing IBMS accredited undergraduate degrees. Universities, especially those identified having no POCT teaching, will have to evaluate their curriculum in light of these changes, if they wish to remain accredited. One possible source of increased teaching support will be local pathology departments. Whilst the data suggests a lack of POCT training in NHS laboratories for newly qualified BMS, there are some clear examples where POCT training is provided using a range of training situations and experience.

Whilst this is the first study to review the curriculum for POCT in laboratory scientists, earlier research in medical (43) and public health curriculums (44) have also reviewed POCT and also found a lack of POCT teaching despite the increased use of POCT in their fields.

## 5.1 Limitations

One of the limitations of this study is that the FOI request only went to NHS laboratories. As private businesses are exempt from the FOI act, they were excluded from the study. Eight of the respondents stated that their pathology services were run by private laboratories and so did not provide any data. These 8 responses were counted amongst the unusable returns. Another limitation is that the information provided depends on the respondent’s interpretation of the request.

The FOI acts also allow respondents not to provide the information if it breaches conditions of the acts, such as cost. Two of the respondents used exemptions explicitly and several more stated that they didn’t hold the information in response to individual questions.

5.2 Conclusion

In Summary, it appears that the amount of POCT taught in UK universities is extremely variable and that in the majority of universities it is taught in generic rather than specific terms.

For UK laboratory training in POCT, the trend is to provide just enough training to meet the HCPC standards, with many laboratories having no rotations in POCT for practical work experience.

The aim of this study was to get the baseline level of POCT teaching and training for laboratory scientists in the UK. This was done objectively and identified a need to further develop POCT teaching and training especially in light of changes in the QAA benchmark statement and increased endorsement in government guidelines and reports.

Further research is needed to see the impact of both an increased use of POCT and the QAA benchmark changes, in the training and teaching of POCT to students and trainees in the UK .

6 **Acknowledgements**

7 **Conflict of Interest**

None

# 8 References

1. Medicines and Healthcare Products Regulatory Agency (MHRA) [In vitro diagnostic point-of-care test devices](https://assets.publishing.service.gov.uk/government/uploads/system/uploads/attachment_data/file/371800/In_vitro_diagnostic_point-of-care_test_devices.pdf) (2021) available at [https://www.gov.uk/government/publications/in-vitro-diagnostic-point-of-care-testdevices](https://www.gov.uk/government/publications/in-vitro-diagnostic-point-of-care-test-devices) [Cited June 29, 2021].
2. Vashist SK. Point-of-Care Diagnostics: Recent Advances and Trends. Biosensors.

2017;7(4). <https://doi.org/10.3390/bios7040062>

3. Park HD. Current Status of Clinical Application of Point-of-Care Testing. Archives of pathology & laboratory medicine. 2021;145(2):168-75.

<https://doi.org/10.5858/arpa.2020-0112-RA>

4. Welsh Government, Healthcare Science in NHS Wales, Looking forward. Available at [https://gov.wales/sites/default/files/publications/2019-03/healthcare-science-in-nhswales.pdf](https://gov.wales/sites/default/files/publications/2019-03/healthcare-science-in-nhs-wales.pdf) [Cited July 2nd, 2021].
5. Welsh Government, Pathology Statement of Intent (2019) available at <https://gov.wales/written-statement-precision-medicine> [Cited July 2nd, 2021].
6. Scottish government, Driving improvement, delivering results: healthcare science national delivery plan 2015-2020 (2015) [https://www.gov.scot/publications/](https://www.gov.scot/publications/driving-improvement-delivering-results-scottish-healthcare-science-national-delivery-plan-20152020/) [Cited July

2nd, 2021].

7. NHS England, NHS Long term plan (2019) available at <https://www.longtermplan.nhs.uk/publication/nhs-long-term-plan/> [Cited July 2nd, 2021].
8. NHS England, Science in Healthcare: Delivering the NHS long term plan – The Chief scientific Officer’s strategy (2020) available at [https://www.england.nhs.uk/](https://www.england.nhs.uk/wp-content/uploads/2020/03/science-in-healthcare-delivering-the-nhs-long-term-plan.pdf) [Cited July

2nd, 2021].

9. Institute of Biomedical Sciences (IBMS) available athttps://www.ibms.org/ [Cited June

29, 2021].

10. Health and Care Professions Council (HCPC) Standards of proficiency for biomedical scientists (2014) available at [https://www.hcpc-uk.org/standards/](https://www.hcpc-uk.org/standards/standards-of-proficiency/biomedical-scientists/) [Cited June 29, 2021].
11. Quality Assurance Agency (QAA) Subject Benchmark Statement, biomedical sciences

(2019) available at [https://www.qaa.ac.uk/](https://www.qaa.ac.uk/docs/qaa/subject-benchmark-statements/subject-benchmark-statement-biomedical-sciences.pdf?sfvrsn=2bf2c881_4) [Cited June 29, 2021].

12. Institute of Biomedical Science (IBMS) Criteria and Requirements for the Accreditation and Re-accreditation of BSc (Hons) in Biomedical Science (2017) available at [https://www.ibms.org/resources/](https://www.ibms.org/resources/documents/criteria-and-requirements-for-the-accreditation-and-re/) [Cited June 29, 2021].
13. Quality Assurance Agency (QAA), Subject Benchmark Statement, biomedical sciences

(2015) available at [https://www.ibms.org/](https://www.ibms.org/resources/documents/qaa-subject-benchmark-statement-biomedical-sciences/) [Cited July 2nd, 2021].

14. Gosling D, Moon J. How to use learning outcomes and assessment criteria. London:

SEEC; 2002.

15. Jenkins A, Unwin, D.. How to write learning outcomes. 2001. Available from:

<https://www.ubalt.edu/> [Cited July 2nd, 2021].

16. Adam S. A consideration of the nature, role, application and implications for European education of employing ‘learning outcomes’ at the local, national and international levels. University of Westminster. 2004 Jun.
17. Moon J. The module and programme development handbook: A practical guide to linking levels, outcomes and assessment criteria. Routledge; 2003 Dec 16.
18. Harden RM. AMEE Guide No. 14: Outcome-based education: Part 1-An introduction to outcome-based education. Medical teacher. 1999;21(1):7–14.
19. Kennedy D, Hyland Á, Ryan N. Writing and Using Learning Outcomes: A Practical Guide. 2007. Available at [https://www.fibaa.org/](https://www.fibaa.org/fileadmin/uploads/content_uploads/Writing_and_Using_Learning_Outcomes_01.pdf#:~:text=Implementing%20Bologna%20in%20your%20institution%20C%203.4-1%20Using,as%20the%20QAA%20%28Quality%20Assurance%20Agency%20for%20Higher) [cited July 2nd, 2021].
20. Mahajan M, Singh MK. Importance and benefits of learning outcomes. IOSR Journal of

Humanities and Social Science. 2017;22(03):65-7.

21. Prideaux D. The emperor's new clothes: from objectives to outcomes. Medical education. 2000;34(3):168-9.
22. Schoepp K. The state of course learning outcomes at leading universities. Studies in Higher

Education. 2019;44(4):615-27.

23. Chatterjee D, Corral J. How to write well-defined learning objectives. The journal of education in perioperative medicine: JEPM. 2017 Oct;19(4).
24. Carr JW, Hardin S. The key to effective assessment: Writing measurable student learning outcomes. Recreational Sports Journal. 2010 Oct;34(2):138-44.
25. Wittmann-Price RA, Fasolka BJ. Objectives and outcomes: The fundamental difference.

Nursing education perspectives. 2010 Jul 1;31(4):233-6.

26. Bloom BS. Taxonomy of educational objectives: The classification of educational goals. Cognitive domain. 1956.
27. Krathwohl DR. A revision of Bloom's taxonomy: An overview. Theory into practice. 2002

Nov 1;41(4):212-8.

28. Stanny CJ. Reevaluating Bloom’s Taxonomy: What measurable verbs can and cannot say

about student learning. Education Sciences. 2016 Dec;6(4):37.

29. Newton PM, Da Silva A, Peters LG. A Pragmatic Master List of Action Verbs for Bloom's

Taxonomy. Frontiers in Education. 2020;5(107).

30. UK government, How to make a freedom of information (FOI) request available at <https://www.gov.uk/make-a-freedom-of-information-request> [Cited July 2nd, 2021].
31. Fowler AJ, Agha RA, Camm CF, Littlejohns P. The UK Freedom of Information Act (2000) in healthcare research: a systematic review. BMJ Open. 2013;3(11):e002967– e002967.
32. Knight J, Stead AP, Geyton TO. Comparing the academic performance of graduate-entry and undergraduate medical students at a UK medical school. Education for health (Abingdon, England). 2017;30(1):75–8.
33. Phillips AW, Friedman BT, Utrankar A, Ta AQ, Reddy ST, Durning SJ. Surveys of health professions trainees: prevalence, response rates, and predictive factors to guide researchers. Academic Medicine. 2017 Feb 1;92(2):222-8.
34. Arulrajah P, Steele S. UK medical education on human trafficking: assessing uptake of the opportunity to shape awareness, safeguarding and referral in the curriculum. BMC medical education. 2018 Dec;18(1):1-7.
35. Ayton A, Ibrahim A. Does UK medical education provide doctors with sufficient skills and knowledge to manage patients with eating disorders safely?. Postgraduate medical journal.

2018 Jul 1;94(1113):374-80.

36. Copeland C, Barron DT. "Delirium: An essential component in undergraduate training?". Nurse education today. 2020;85:104211.
37. Ormerod R. The history and ideas of pragmatism. Journal of the Operational Research

Society. 2006 Aug;57(8):892-909.

38. Duram L. Pragmatic Study. Vol. 2, Encyclopedia of Research Design. 2010. p. 1072–4.
39. Institute of Biomedical Sciences, IBMS accredited undergraduate degree Courses (2018) [https://www.ibms.org/](https://www.ibms.org/resources/documents/ibms-accredited-undergraduate-degree-coursesa/) [Cited July 2nd, 2021].
40. The rise of POCT IBMS available at [https://thebiomedicalscientist.net/](https://thebiomedicalscientist.net/science/rise-poct#:~:text=New%20IBMS%20POCT%20qualification%20The%20new%20IBMS%20POCT,with%20Ulster%20University%2C%20which%20will%20host%20it%20online) [Cited Oct 2nd,

2021].

41. Price CP, St John A. Will COVID-19 be the coming of age for point-of-care testing?.

BMJ Innovations. 2021 Jan 1;7(1).

42. Lewis T, Wood M, Myers M. Pathology GIRFT Programme National Specialty Report

GIRFT; 2021 [Cited Jan 20^th^, 2022]

43. Mavenyengwa RT, Nyamayaro T. Developing a curriculum for health professional students on point of care testing for medical diagnosis. International journal of medical education. 2016;7:265-6.
44. Kost GJ, Zadran A, Zadran L, Ventura I. Point-Of-Care Testing Curriculum and Accreditation for Public Health—Enabling Preparedness, Response, and Higher

Standards of Care at Points of Need. Frontiers in public health. 2019;6.

Supporting information

Appendix 1. Examples of definitions of a learning outcome

| **Definition** | **Source** |
| --- | --- |
| *Learning outcomes are statements of what is expected that the student will be able to do as a result of learning the activity.* | Jenkins and Unwin, 2001  (15) |

| *Learning outcome: a statement of what a learner is expected to know, understand and/or be able to demonstrate at the end of a period of learning”.* | Gosling and Moon, 2001  (14) |
| --- | --- |
| *A learning outcome is a written statement of what the successful student/learner is expected to be able to do at the end of the module/course unit or qualification.* | Adam, 2004 (16) |
| *A learning outcome is a statement of what a learner is expected to know, understand and be able to do at the end of a period of learning and of how that learning is to be demonstrated”.* | Moon, 2002 (17) |
| *In outcome-based education, the outcomes agreed for in the curriculum guide what is taught and what is assessed.* | Harden, Crosby & Davis  1999 (18) |
| *Learning outcomes are statements of what a learner is expected to know, understand and/or be able to demonstrate after completion of a process of learning* | Kennedy, Hyland & Davis  2007 (19) |

543

544	Appendix 2. Suggested structures of course learning outcomes.

545

| Structure | Reference | Good LO example |
| --- | --- | --- |
| Begin with a consistent short stem: such as “Students will be able to” or “At course completion students “ | Schoepp  2017 (22) | Students can critically review the methodology of a research |

| State the desired student performance/behaviour using concrete action, or operational, verbs such as create, apply, interpret, describe, identify, categorize |  | study published in a marketing journal. |
| --- | --- | --- |
| SMART objectives  **S**pecific: What action will be performed and by whom?  **M**easurable: How will success be measured? Objectives should quantify the amount of change expected.  **A**chievable: Can this objective be achieved within a given time frame and with available resources? **R**elevant: Are the objectives aligned with the instructional method and assessment?  **T**ime-Bound: When will this objective be achieved? Objectives should provide a time frame indicating when the objective will be met. | Chatterjee and Corral 2017 (23) | Upon completion of the difficult airway workshop, participants should be able to formulate an accurate algorithm for the management of an obese adult patient with inadequate  face mask ventilation, according to the American Society of Anesthesiologists difficult airway algorithm |
| The ABCD method  A = Audience is the target group of the LO. The learning outcomes statement would begin something like: “The students will . . .” or “The participants will .  . .” | Carr and  Hardin  2010 (24) | In response to four scenarios, each student staff member will correctly articulate the steps of  the Emergency Action Plan procedures as outlined in the student staff manual. |
| B = Behaviour. The behaviour is the action that will be performed by the “audience.” The action word is the “verb” in the sentence. C = Condition. A condition is the circumstance under which the behaviour will take place examples include   “by the end of the second session or by the start of the spring semester.”   D = Degree is the element of the learning outcome that is measurable. It basically describes how well a behaviour must be accomplished. These include (“85% correct” or “with fewer than three errors”). |  |  |
| The ABC method   A = antecedent or the learning activity; B = behaviour or the skill or knowledge being demonstrated C = the criterion or the degree of acceptable performance. | WhitmannPrice and  Fasolka  2010 (25) | (A) By the end of this session the learner will be able to: (B) Demonstrate sterile Foley catheter insertion (C) 100% of the time in clinical |
| Begin each learning outcome with an active verb, followed by the object of the verb followed by a phrase that gives the context. | Kennedy et  al 2007  (19) | Classify reactions as  exothermic and endothermic. |

546

547	Appendix 3. Count of verbs used in learning outcomes

| **Row Labels** | **Count of verbs** |
| --- | --- |

explore	1 formulate	1 Highlight	1 execute	1 undertake	1 appraise	1 benchmark	1 outline	1 conduct	1 plan	1 contribute	1 provide	1 criticise	1 receive	1 design	1 record	1 arrange	1 relate	1 consider	1 review	1 decide	1 satisfy	1 compare	1 select	1 differentiate	1 summarise	1 cost	1 theoretically underpin	1 appreciate	2 uphold	2 reflect	2 know	2 comply	2 identify	2 respect	2 acquire	3 compose	3 justify	3 use	3 aware	4 communicate	4 work safely	4 apply	4 perform	5 develop	6 know	7

Appendix 4

| **Grand Total** | **192** |
| --- | --- |

| recognise | 7 |
| --- | --- |
| analyse | 8 |
| describe | 9 |
| interpret | 10 |
| discuss | 10 |
| demonstrate | 11 |
| explain | 12 |
| evaluate | 13 |
| understand | 24 |

count of action verb by level of blooms taxonomy

| **Row Labels** | **Count of Masterlist (time on source lists)** |
| --- | --- |
| **Analysis** | **12** |
| Analyse | 8 |
| Appraise | 1 |
| Compare | 1 |
| Differentiate | 1 |
| Outline | 1 |
| **Application** | **33** |
| Apply | 4 |
| Conduct | 1 |
| Contribute | 1 |
| Criticise | 1 |
| Demonstrate | 11 |
| Execute | 1 |
| Interpret | 10 |
| Provide | 1 |
| Use | 3 |
| **Avoid** | **42** |
| Appreciate | 2 |
| ARRANGE | 1 |
| Aware | 5 |
| know | 9 |
| RELATE | 1 |
| SELECT | 1 |
| Understand | 23 |
| **Comprehension** | **30** |

Discuss	10

| Develop | 6 |
| --- | --- |
| Evaluate | 13 |
| Justify | 3 |
| **knowledge** | **23** |
| Acquire | 3 |
| Describe | 9 |
| highlight | 1 |
| Identify | 2 |
| Recognise | 7 |
| Record | 1 |
| **not on list** | **14** |
| benchmark | 1 |
| Cost | 1 |
| receive | 1 |
| Respect | 2 |
| satisfy | 1 |
| theoretically underpin | 1 |
| undertake | 1 |
| uphold | 2 |
| work safely | 4 |
| **Synthesis** | **15** |
| Communicate | 4 |
| Comply | 2 |
| Compose | 3 |
| Consider | 1 |
| Design | 1 |
| Formulate | 1 |
| Plan | 1 |
| Reflect | 2 |
| **SYNTHESIS 4, EVALUATION 4** | **1** |
| Decide | 1 |
| **Grand Total** | **192** |

| Explain | 12 |
| --- | --- |
| Explore | 1 |
| Perform | 5 |
| Review | 1 |
| Summarise | 1 |
| **Evaluation** | **22** |

Appendix 5. Response to question 2 of survey 2, regarding types of evidence to meet health and care professions council standards

14.16 and 14.26

| Evidence Type | Whole laboratory response | Microbiology | Blood sciences | Haematology | Histopathology | Specialist | Imm |
| --- | --- | --- | --- | --- | --- | --- | --- |
| Annotated work | 18 | 11.0 | 2 | 6 | 6 | 0 |  |
| Audit | 13 | 3.0 | 2 | 2 | 5 | 0 |  |
| Case study | 9 | 5.0 | 2 | 2 | 3 | 0 |  |
| Competency assessment | 55 | 10.0 | 9 | 8 | 12 | 1 |  |
| Essay/review | 23 | 9.0 | 3 | 11 | 8 | 2 |  |
| Journal club | 0 | 1.0 | 0 | 0 | 0 | 0 |  |
| Lecture/tutorial | 9 | 5.0 | 1 | 3 | 1 | 1 |  |
| MDT | 1 | 0.0 | 0 | 0 | 0 | 0 |  |
| Personal statement | 5 | 0.0 | 0 | 0 | 1 | 0 |  |
| Practical | 12 | 4.0 | 3 | 5 | 2 | 1 |  |
| Questions & Answer | 35 | 13.0 | 9 | 10 | 11 | 1 |  |
| Reflective statement | 61 | 22.0 | 11 | 16 | 19 | 4 |  |
| Self directed learning | 3 | 1.0 | 0 | 1 | 0 | 0 |  |
| University | 3 | 0.0 | 0 | 0 | 0 | 1 |  |
| Witness statement | 30 | 11.0 | 6 | 6 | 11 | 1 |  |

555

556

Appendix 6. Response to question 3 of survey 2 regarding hours trained in point of care testing

| **Laboratory** | **Useable responses** | **0 hours**  **teaching**  **(n)** | **Unknown**  **hours teaching**  **(n)** | **Range**  **of hours** | **Mean (hours)** |
| --- | --- | --- | --- | --- | --- |
| whole trust response | 81 | 21 | 13 | 0-150 | 17.6 |
| Specialist laboratories | 7 | 0 | 2 | 1-7 | 3.4 |
| Histology/cytology | 39 | 19 | 4 | 0-15 | 2.7 |
| Immunology | 7 | 4 | 2 | 0-7 | 1.4 |
| Haematology | 29 | 4 | 6 | 0-37.5 | 6.6 |
| Biochemistry | 24 | 2 | 2 | 0-450 | 76.2 |
| Blood sciences | 21 | 1 | 1 | 0-112.5 | 17 |
| Microbiology/virology | 46 | 15 | 7 | 0-15 | 2.3 |

557

558	Appendix 7. Response to question 4 of survey 2, regarding training methods for point of care in clinical laboratories.

| **Laboratory** | **Useable responses** | **No training** | **Practical training** | **lecture/ tutorial** | **shadowing**  **/ tour** | **Self directed** | **E-learning** | **University** |
| --- | --- | --- | --- | --- | --- | --- | --- | --- |
| Whole trust  response | 77 | 12 | 42 | 44 | 21 | 3 | 4 | 5 |
| Specialist laboratories | 7 | 0 | 6 | 5 | 3 | 1 | 0 | 1 |
| Histology/ cytology | 27 | 6 | 9 | 14 | 8 | 3 | 1 | 0 |
| Immunology | 5 | 3 | 2 | 2 | 0 | 0 | 0 | 0 |
| Haematology | 27 | 4 | 12 | 16 | 6 | 2 | 1 | 0 |
| Biochemistry | 23 | 1 | 20 | 13 | 5 | 0 | 2 | 0 |
| Blood sciences | 21 | 1 | 14 | 14 | 9 | 1 | 0 | 0 |
| Microbiology/ virology | 38 | 7 | 14 | 22 | 9 | 3 | 1 | 0 |

559

560	Appendix 8. Response to question 5 of survey 2, regarding rotations into a point of care testing section/department.

| **Laboratory** | **Useable results** | **Range of hours** | **No**  **POCT**  **rotation** | **N/A** | **Unknown** | **Mean** |
| --- | --- | --- | --- | --- | --- | --- |
| whole trust response | 90 | 0-450 | 56 | 7 | 10 | 18.2 |
| Specialist laboratories | 12 | 0-7.5 | 7 | 4 | 0 | 0.94 |
| Histology/cytology | 38 | 0-3.75 | 28 | 7 | 0 | 0.73 |
| Immunology | 8 | 0-7.5 | 5 | 2 | 0 | 1.25 |
| Haematology | 26 | 0-30 | 22 | 0 | 0 | 2.2 |
| Biochemistry | 23 | 0-450 | 7 | 0 | 2 | 86.7 |
| Blood sciences | 16 | 0-112.5 | 6 | 0 | 3 | 17.3 |
| Microbiology/virology | 40 | 0-15 | 23 | 6 | 4 | 1.6 |

561

POCT: point of care testing

Revised Manuscript with Track Changes

**1**

**|**

Page

**What is the scope of teaching and training of undergraduate students**

**and trainees in Point of Care Testing in UK universities and hospital**

**laboratories?**

**Lee Peters***

**1**

**,**

**Ana Sergio Da**

**Silva**

**1**

**,**

**Philip M**

**ark**

**~~.~~**

**Newton**

**1**

1

1

Research in Health Professions Education, Swansea University Medical School, Swansea

2

University, Swansea, Wales, United Kingdom SA2 8PP

3

***Correspondence:**

4

Corresponding

Author

5

@swansea.ac.uk

141750

6

**Keywords:**

Laboratory training

,

B

iomedical science

,

Freedom of information request, HCPC

,

7

POCT

8

**1**

**Abstract**

9

Point of Care Testing (POCT) is an analytical test performed by a healthcare professional outside

10

of a conventional laboratory. The global POCT market was valued at

US$ 23.16

billion

in 2016

11

and is forecasted to grow

to US$ 36.96 billion in 2021

. This upward trend for POCT has increased

12

workload for pathology departments who manage POCT. This research aims to characterize and

13

analyse the teaching and training of POCT at

United Kingdom

(

UK

)

universities on Institute of

14

Biomedical Science (IBMS) accredited biomedical science degrees, and at UK hospital

15

laboratories.

16

A f~~F~~reedom of i~~I~~nformation (FOI) request was sent in 2018 to all 52 UK universities with an accredited IBMS Biomedical science degree to request information on teaching of POCT, with a 100% response rate. Further FOI requests were sent to all NHS hospital pathology departments in the UK, regarding POCT training provided to trainee Biomedical scientists, with a 97% response

rate.

Twelve~~12~~ of the degrees contained no POCT teaching, with a further 9 having no specific POCT teaching. Sixty-six~~66~~ laboratories confirmed that there was ~~replied they had~~ no POCT training.

The university teaching hours varied between 0~~1~~ and 35 hours. The median time spent teaching POCT at university was ~~18.5~~2 hours. The laboratory teaching hours varied between 0 and 450 hours The ~~average~~ median time spent teaching POCT in hospital laboratories~~s~~ was ~~16.5~~3 hours.

A content analysis of the learning outcomes provided by 29 universities showed that only 61% (84/137) were measurable and 26~~31~~% (36/137) of the learning outcomes used action verbs that have previously been listed to be avoided in learning outcome writing. Only 9%~~13~~(13/137) of outcomes ~~(9%)~~ specifically described POCT, with 8 of these being measurable.

The findings demonstrate that although this is a commonly required skill for biomedical scientists there is a clear lack of POCT teaching and training in the UK.~~, both in quality and quantity,~~ T~~t~~o meet the new QAA guidelines, but most importantly to ensure the workforce is fit for the needs of the current healthcare system, the quality and quantity of POCT teaching and training needs to improve.

## 2 Introduction

The

Un

ited Kingdom (

UK

)

Medicines and Healthcare

p

~~P~~

roducts Regulatory Agency (MHRA)

38

define Point of Care Testing (POCT) as any analytical test performed for a patient by a healthcare

39

professional outside

of

the conventional laboratory setting (

1

)

. Other terms co

mmonly used to

40

describe POCT include

~~N~~

n

ear

~~P~~

p

atient

~~T~~

t

esting (NPT)

and be

dside testing

.

41

POCT use is increasing, with t

he global POCT market expected to grow from US$ 23.16

billion

42

in 2016 to US$ 36.96 billion in 2021

(

2

).

T

his increasing demand has been

attributed to increasing

43

clinical demand, heavy industry promotion, short turnaround time for results, economical and

44

practical factors, and advancements in technology (

3

)

.

Examples of POCT include Gluc

ose meters,

45

Pregnancy tests,

INR

and SARS

-

C

oV

-

2

.

The g

lobal pandemic has exacerbated demands for

46

POCT.

To meet this growing demand, laboratory staff will need to be trained to

support and

deliver

47

this service.

48

The growth and development of

POCT has been identified in key UK government healthcare

49

strateg

ies

~~y~~

. An example

,

is Looking forward: Healthcare science in NHS Wales (

4

)

. This

50

document published by the Welsh government gives POCT in the community as an example

of

a

51

new role for healthcare scientists. In 2019 the Welsh government also published the Pathol

ogy

52

statement of intent (

5

. This document lists POCT

)

as a

pathology

specialty

and that it is one of the

53

fields that

is

*“... rapidly evolving, driven by a reduction in costs, increased connectivity and*

54

*technological innovation”.*

The statement of

i

ntent als

o discusses how POCT will be part of the

55

service design of pathology in Wales. The Scottish healthcare science national delivery plan 2015

-

56

2020 (

6

, lists POCT as one of the five service improvement programmes that

)

will

*“… deliver*

57

*high*

*-*

*quality, sustainable*

*health and care services for Scotland…”*

*.*

The NHS England long

-

term

58

plan (

7

)

discusses the use of POCT as a method to prevent hospital admissions

.

T

he Science in

59

**Formatted:**

Font:

Not

Italic

care pathways.

### 2.1 Biomedical science education in UK

Biomedical scientists (BMS). Biomedical scientist is the protected title of a laboratory scientist that has met the HCPC BMS standards of proficiency. ~~Biomedical science staff mostly work in~~

routes to HCPC registration for ~~biomedical scientists.~~BMS

Healthcare: delivering the

long

-

term

plan report (

8

)

from the Chief scientific officer of Engl

and,

list

s

POCT as an emerging technology that will shape healthcare science and a key component in

The Health and Care professions Council (HCPC) is the regulator of 15 health and care professions

in the U

K. The professions include

~~Clinical scientists,~~

P

hysiotherapists,

R

adiographers and

~~clinical laboratories~~

~~identifying~~

~~diseases and by analysing samples from patients~~

.

Fig

1

shows the

Fig

1

The

Four

~~4~~

routes to Health and Care Professions Council

)

HCPC

(

registration for

HCPC registration

adapted from information from Institute of Biomedical Science on becoming

(

The

Institute of Biomedical Science (

IBMS

)

Certificate of competence (COC) is a qualification

that demonstrates the student/trainee meets the HCPC standards of proficiency for registration as

a

~~biomedical scientist~~

BMS

(

10

)

. In routes one and two, s

tudents will undertake an IBMS

accredited undergraduate degree and

~~complete~~

laboratory training either during or after completion

of the degree. Route 3 is for students that don’t have an accredited degree and require educational

**Formatted:**

Space

After:

0

pt

**Formatted:**

Space

Before:

0

pt,

After:

0

pt

**Formatted:**

Superscript

biomedical scientists ~~(adapted from information from IBMS on becoming HCPC registered~~

~~(9)).~~‘Top-up’ education is supplementary education to meet the HCPC requirements. The equivalence process is for laboratory scientists that work at the level of BMS, that don’t have

HCPC registered (9)).

1. Certificate of Competence (COC). Biomedical Scientists (BMS).

‘Top

-

up’s’

,

to ensure they

meet the educational requirements

,

and

clinical

laboratory training.

82

Route 4 is an equivalence route to registration. This route is not part of this research due to low

83

numbers and a non

-

standard

ised

training

program

.

84

**2.2**

**IBMS degree accreditation and the UK**

**Quality Assurance Agency (QAA)**

85

Undergraduate d

egree accreditation by the IBMS ensures the degree covers the specified subjects

,

86

in specific depth,

to meet the HCPC standards of proficiency for

~~B~~

~~iomedical scientists~~

BM

S

.

87

Degrees are only accredited if they

incorporate the requirements detailed in the

Quality Assurance

88

Agency for Higher Education (QAA)

subject benchmarking for Biomedical

science

(

11

)

.

89

Accreditation is normally awarded for five

years

, after which t

he university must apply for re

-

90

~~accreditation~~

~~of the degree (~~

~~12~~

~~)~~

.

The accreditation process also looks at other aspects of the

91

program

, such as,

program

delivery

~~, staff development, employer liaison~~

and placement

92

opportunities

(12)

.

93

The QAA is an independent body that monitors and advises on standards

and quality in UK higher

94

education.

QAA benchmark statements define the academic standards that can be expected of a

95

graduate , in terms of what they might know, do and understand at the end of their studies and

96

describe the nature of the subject (

11

.

)

Be

nchmark statements are produced by a group of subject

97

specialists drawn from, and acting on behalf of, the subject community.

Changes are proposed and

98

~~reviewed by the benchmark review group~~

. The QAA initiates regular reviews of their content

, five

99

~~years~~

~~after first publication, and every seven years subsequently~~

, or in response to significant

100

changes in the discipline (

11

)

.

They don’t provide

a

national curriculum for a subject but provide

101

general guidance for writing the learning outcomes of the course.

102

POCT

is referenced twice in the HCPC standards of proficiency for

~~Biomedical scientists~~

BMS

:

103

**Formatted:**

Line

spacing:

Double

1. Standard 14.16, know the extent of the role and responsibility of the laboratory with respect to the quality management of hospital, primary care and community-based laboratory services for ~~near-patient testing~~NPT and non-invasive techniques.
2. Standard 14.26, be able to use standard operating procedures for analyses including point of care in vitro diagnostic devices

Students or trainees undertaking the COC must meet all the standards of proficiency, including the standards shown in table 1. To become IBMS accredited, undergraduate courses only have to meet the QAA benchmarking statement.

The QAA benchmark statement released late 2019 (11) now includes ~~near-patient testing~~NPT under the clinical Chemistry requirements. All universities seeking IBMS accreditation or reaccreditation post 2019 will have to evidence how they meet the new benchmark statement. ~~Near~~ ~~patient testing~~NPT has been added to the benchmark statement *“the principles and applications of biochemical investigations used for screening, diagnosis, treatment and monitoring of disease, including near-patient testing”.* POCT has been added *“in light of recent advances in practice”.*

**Formatted:**

Line

spacing:

Double

(11)

POCT was not specifically mentioned in the previous QAA Biomedical science statement (13). This research was conducted before the new QAA benchmark statement was released in late 2019. Therefore, universities who responded to the FOI request were accredited under the 2015 benchmark statement.

### 2.3 Learning outcomes

Learning outcomes (LOs) are a statement of what a learner is expected to know and be able to demonstrate by assessment after learning. Gosling and Moon (14 p.11) state that;

*“Well written learning outcomes provide a means of mapping the content of a curriculum - – for example, to see how they reflect benchmark statements, which of the key skills are acquired, where the same skill or content is appearing more than once in the programme, the capabilities the students acquire as they progress through the levels in the programme of study.”*

There appears to be no standard definition of a learning outcome, several different definitions have been suggested, ~~see table 1 below~~ see appendix 1. Whilst there is no standard definition there is a conserved theme. Kennedy, Hyland & Davis (19) ~~in their review of learning outcomes~~, stated that the various definitions they cited did not differ significantly and it was clear that learning outcomes focus on what the learner has achieved ~~rather than the intentions of the teacher~~ and that learning outcomes focus on what the learner can demonstrate at the end of a learning activity.

| **Definition** | **Source** |  |
| --- | --- | --- |
| *Learning outcomes are statements of what is expected that the student will be able to do as a result of learning the activity.* | Jenkins and Unwin, 2001  (15) |  |
| *Learning outcome: a statement of what a learner is expected to know, understand and/or be able to demonstrate at the end of a period of learning”.* | Gosling and Moon, 2001  (14) |  |
| *A learning outcome is a written statement of what the successful student/learner is expected to be able to do at the end of the module/course unit or qualification.* | Adam, 2004 (16) |  |
| *A learning outcome is a statement of what a learner is expected to know, understand and be able to do at the end of a period of learning and of how that learning is to be demonstrated”.* | Moon, 2002 (17) | |
| *In outcome-based education, the outcomes agreed for in the curriculum guide what is taught and what is assessed.* | Harden, Crosby & Davis  1999 (18) | |
| *Learning outcomes are statements of what a learner is expected to know, understand and/or be able to demonstrate after completion of a process of learning* | Kennedy, Hyland & Davis  2007 (19) | |

Table 1 Some proposed definitions of a learning outcome

Whilst there is no standard definition there is a conserved theme. Kennedy, Hyland & Davis (19) in their review of learning outcomes, stated that the various definitions they cited did not differ significantly and it was clear that learning outcomes focus on what the learner has achieved rather than the intentions of the teacher and that learning outcomes focus on what the learner can demonstrate at the end of a learning activity.

The use of learning outcomes has been reported to have many benefits. They enable internal and external quality assurance to determine how appropriate the curriculum is (14). The learning outcomes allow students to gauge what they can achieve if they participate on a module/course. Mahajan and Sarjit Singh (20) listed a range of perceived benefits that in their opinion included; helping the student choose an appropriate course, give a clear idea to the students of what are they going to learn or achieve at the end of the class before the start of every class, help teachers design

151

152

153

154

| **What is the scope of teaching and training of undergraduate students and trainees in Point of Care Testing in UK universities and hospital laboratories?**  their teaching material more effectively, help teachers select appropriate strategies for teaching and make assessments mapping clear & easy.  Given the importance of LOs throughout the years many theorists have suggested approaches to their design. Through the decades the different approaches have tried to find a balance between | | | |  | |
| --- | --- | --- | --- | --- | --- |
| being measurable but also usefulness and “user-friendly”. Prideaux (21), discusses the use | | | of s g  d |  |  |
| objectives starting with Ralph Tyler’s work in 1949 through to behavioral objectives of the 1960 | | |  |  |  |
| and 70s, which were complex to write and focused on observable behaviors, and then onto learnin | | |  |  |  |
| ~~outcomes or outcomes-based education.~~  ~~Structures described for writing a good learning outcome are described in table 2:~~Some suggeste | | |  |  |  |
| structures for a good learning outcome are described in appendix 2 | | |  |  |  |
| Structure | Reference | Good LO example |  |  |  |
| Begin with a consistent short stem: such as “Students will be able to” or “At course completion students “  State the desired student performance/behavior using concrete action, or operational, verbs such as create, apply, interpret, describe, identify, categorize | Schoepp  2017 (22) | Students can critically | re | view |  |
|  |  | the methodology of a r | es | earch |  |
|  |  | study published in a ma | rk | eting |  |
|  |  | journal. |  |  |  |
| SMART objectives  **S**pecific: What action will be performed and by whom?  **M**easurable: How will success be measured? | Chatterjee and Corral 2017 (23) | Upon completion o | f | the |  |
|  |  | difficult airway wo | rks | hop, |  |
|  |  | participants should be | ab | le to |  |
|  |  | formulate an accurate al | gor | ithm |  |
|  |  |  |  |  |  |
| 9 | | | |  |  |

155

156

157

158

159

160

Objectives should quantify the amount of change

expected.

**A**

chievable: Can this objective be achieved within a

given time frame and with available resources?

**R**

ele

vant: Are the objectives aligned with the

instructional method and assessment?

**T**

ime

-

Bound: When will this objective be achieved?

Objectives should provide a time frame indicating when

the objective will be met.

bese

for the management of an o

adult patient with inade

quate

face

mask

ventila

tion,

according to the Ame

rican

gists

Society of Anesthesiolo

diff

icult airway algorithm

The ABCD method

A = Audience is the target group of the LO. The

learning outcomes statement would begin something

like: “The students will . . .” or “The participants will .

. .”

B = Behavior The behavior is the action that will be

performed by the “audience.” The action word is the

“verb” in the sentence.

C = Condition A condition is the circumstance under

which the behavior will take place examples include

“by the end of the second session or by the start of the

spring semester.”

D = Degree is the element of the learning outcome that

is measurable. It basically describes how well a

Carr

and

Hardin

2010

(24

)

r

In response to four scena

ios,

each student staff member

will

correctly articulate the ste

ps of

the Emergency Action

Plan

procedures as outlined in

the

student staff manual.

161

162

163

164

165

| **What is the scope of teaching and training of undergraduate students and trainees in Point of Care Testing in UK universities and hospital laboratories?** | | | |  | |
| --- | --- | --- | --- | --- | --- |
| behavior must be accomplished. These include (“85% correct” or “with fewer than three errors”). |  |  |  |  |  |
| The ABC method   A = antecedent or the learning activity; B = behavior or the skill or knowledge being demonstrated C = the criterion or the degree of acceptable performance. | WhitmannPrice and  Fasolka  2010 (25) | (A) By the end of this | se | ssion |  |
|  |  | the learner will be able | to | (B)  : |  |
|  |  | Demonstrate sterile |  | Foley |  |
|  |  | catheter insertion (C) 1 | 00 | % of |  |
|  |  | the time in clinical |  |  |  |
| Begin each learning outcome with an active verb, followed by the object of the verb followed by a phrase that gives the context. | Kennedy et  al 2007  (19) | Classify reactions |  | as |  |
|  |  | exothermic and endothe | rm | ic. |  |
|  |  |  |  |  |  |
| Table 2 Suggested structures of course learning outcomes.  Learning outcomes use verbs to describe the expectations of the student. Bloom's Taxonomy, first published in 1956 (26) and revised in 2002 (27), is often used to write LOs. Bloom’s taxonomy classifies thinking skills into six hierarchically organized categories that range from lower-level (know and understand) to higher-order (apply, analyze, evaluate, create) (26). Each of the levels have indicative verbs. Stanny (28), and in our previous work (29), analyzed examples of verb lists on educational websites in America and UK respectively. Both studies found little consistency in the lists, including several verbs that appeared in all levels of the hierarchy suggested by Bloom. We have previously suggested a list of verbs to use at each hierarchical level and a list of verbs to avoid using in writing learning outcomes (29).  11 | | | |  |  |

166

167

168

169

170

171

### 2.4 Freedom of Information (FOI) requests

The UK freedom of information act (2000) and The f~~F~~reedom of i~~I~~nformation a~~A~~ct (FOIA) and f~~F~~reedom of i~~I~~nformation (Scotland) a~~A~~ct (2002) (30) gives the right to access recorded information held by public sector bodies. The FOI act has some limitations about what information is covered by the act, e.g., cost above the allowable threshold, repeat of a previous request or the request is vexatious. To make a FOI request, the organisation must be contacted in writing, e.g., e-mail, letter, online form and~~. The information that is~~ requires~~d to submit a request is; Y~~your name, contact details and detailed description of the information required. The legislation allows 20 working days for a response.

Freedom of Information (FOI) requests have been used as a mechanism to distribute surveys, questionnaires etc. to gather data for research. Fowler et al (31) conducted a systematic review of the use of the UK FOI act to gather data for healthcare research. The 16 studies reviewed covered a range of requests such as litigation, surgical provision and ~~,~~ laboratory provision, ~~pharmacological safety and midwifery discipline~~. ~~The studies reviewed generated 15817 pieces of~~ ~~data.~~ One of the studies included in the review tried to obtain data via a letter and achieved a response rate of 11%. When they made the same request via FOI the response rate rose to 83%. FOI requests have also been used in educational research. Knight, Stead & Geyton (32) used a FOI to look at the academic performance of graduate-entry and undergraduate medical students at a UK medical school.

**Formatted:**

Line

spacing:

Double

~~A survey, via a FOI request, was used as the contact details of pathology departments are not~~ ~~centrally available, or easily found and so getting the survey to the right people would have been~~ ~~problematic and potentially reduced the response rate. A FOI request was therefore used a~~

~~pragmatic tool to gather the information required~~

~~.~~

Phillips et al

(33)

reported a response rate

194

between 26.6% and 100%, with a mean of 71.3% (SD 19.5%), when they looked at survey

195

response rates in three education journals. The

y compared this to other reported response rates;

196

United states government surveys

(

~

70

%),

and other surveys across Europe (50

-

60

%

).

Health

197

profession education research using FOI requests have shown a response rate of 97%

,

93

%,

83

%

198

(

Arulrajah and Steele

(34)

,

Ayt

o

n and Ibrahim

(35)

,

Copeland and Barron

(36)

)

respectively.

199

The research presented here is a pragmatic approach to answer the research questions to inform

200

further research into the

~~biomedical scientist~~

BMS

curriculum

and laboratory training

with

regards

201

to POCT teaching and training.

Richard Omerod described

pragmatism as

*“a philosophical*

202

*doctrine that can be traced back to the academic sceptics of classical antiquity who denied the*

203

*possibility of achieving authentic knowledge regarding the real t*

*ruth and taught that we must make*

204

*do with plausible information adequate to the needs of practice”*

(

37

. Pragmatic research

)

aims to

205

~~be useful and~~

~~focuses on real world problems (~~

~~38~~

~~).~~

206

~~1.~~

The current state of POC

T

teaching in universities and clinical laborator

s

ies i

207

unknown

and t

his research seeks to answer the following research questions:

208

~~2.~~

1.

How much POCT is taught in UK accredited undergraduate Biomedical science

209

degrees pre change in QAA benchmark statement?

210

~~3.~~

2.

~~What~~

How

POCT is taught in UK accredited

undergraduate Biomedical science

211

degrees pre change in QAA benchmark statement?

212

~~4.~~

3.

How much POCT is taught in UK pathology laboratories pre change in QAA

213

benchmark statement?

214

~~5.~~

4.

~~What~~

How

POCT is taught in UK pathology laboratories

pre

change in QAA

215

benchmark sta

tement?

216

**Formatted:**

Indent:

Left:

",

0.5

No

bullets

or

numbering

**3	Materials and Methods**

A survey was sent to all UK universities offering IBMS accredited Biomedical science degrees in May 2018, with second survey sent to all the NHS trusts/Health board laboratories in the UK in October/November 2018. The Surveys were sent using a FOI request. ~~Table 3 gives the wording~~ ~~of each of the surveys sent via freedom of information requests.~~The wording for each survey is provided in Table 1~~3~~.

| **Survey 1 (n=52)** | **Survey 2 (n=179)**  For each pathology discipline in your  Trust/Health Board (i.e. Microbiology,  Haematology, Blood transfusion,  Biochemistry, Histopathology etc…); |
| --- | --- |
| 1. Please could you list all your ~~IBMS~~ Institute of Biomedical Science (IBMS) accredited courses your institution currently run? | 1. On average how many trainee Biomedical scientist/university biomedical science placement students do you have a year? |
| 2. Approximately how many hours of teaching time is dedicated to Point of Care Testing/ Near patient testing on each of your IBMS accredited courses? | 2. What types of evidence are used by your students/trainees to meet the ~~HCPC~~ Health and Care Professions Council (HCPC) Standards of proficiency for biomedical scientists for standards 14.26 and 14.16 in the |

pathology laboratories train their trainee ~~Biomedical scientists~~BMS on POCT including how they met the relevant HCPC standards. ~~As described in the introduction, this information will cover~~ ~~trainees/students going through pathways 1-3 to HCPC registration. The first survey will cover~~ ~~pathways 1 and 2 as students /trainees going down these pathways will have completed an~~ ~~accredited BMS degree, whilst the second survey covers 1-3 as all students/trainees going through~~ ~~these pathways will have to have a clinical laboratory placement and achieve the COC~~ The separate surveys were done in order to assess the different avenues of HCPC registration of trainees/students (refer to Fig 1). The participants in survey one complete an IBMS accredited degree (pathways 1 and 2) whilst those of survey 2 achieve a COC during a clinical placement or training positio. The second survey asked the laboratories to provide information for all relevant disciplines in their hospital trust or health board. This was to capture practices per pathology discipline, if there was no pan pathology approach to training.

**Formatted:**

Line

spacing:

Double

The surveys were sent a few months apart due to the large number of requests being sent out and to allow time to deal with queries.

### 3.1 Selection process

A list of UK-based IBMS-accredited degrees was obtained from the IBMS website (39). Only UK universities were selected as the aim of the research was to identify POCT training in the UK. This resulted in 52 university FOI requests.

Despite an extensive search, there is no list of pathology laboratories in the UK. Pathology laboratories are the main provider of employment of ~~Biomedical scientists~~BMS. Therefore, an FOI request was sent to every NHS acute trust or Health board in the UK. To identify these, the websites for NHS England, Wales, Scotland and Northern Ireland were searched . Additionally, the Public health and Blood transfusion trusts for each nation were also added to the list. The trusts and Health boards were selected as they host pathology as part of their organsiation. This ~~resulted in~~identified 179 institutions (trusts/healthboards) where FOI requests could be sent.

The FOI requests were sent to each university/Trust/Health board dependent on the instructions on their internet site.

### 3.2 Data Analysis

All responses were recorded in Excel for analysis. Each response was anonymised and given a unique identifier to avoid any potential reputational damage to the universities and laboratories. Response rate, response turnaround time, teaching and training hours (range, ~~and~~ median~~an~~ and interquartile range) were calculated. If range of training hours was provided, then the maximum value was used.

**Formatted:**

Line

spacing:

Double

Learning outcomes (LO) were reviewed and assessed against the following criteria:

1. Was the LO measurable?
2. Does the LO specifically describe POCT?
3. Does the LO have multiple outcomes?
4. Is the action verb used on the recommended/avoid list as described by Newton et al (29)?
5. What level of blooms taxonomy would the verb be mapped to?

The LO were assessed by at least one author and then reviewed by the other two authors. Any changes required agreement by all three authors~~were agreed by all 3 authors~~.

To assign the verb to a level of blooms taxonomy, the authors used the supplementary data from Newton et al (29). The supplementary data includes the Verb lists from 47 UK higher education institutions. Verbs were assigned to the level where it appeared in the majority of these lists.

~~To assign the verb to a level of blooms taxonomy, the supplementary data from Newton et al, Da~~ ~~Silva & Peters (29) was used. Verbs were assigned to the level where it appeared in the majority~~ ~~of lists searched during that study.~~

For training hours, a day was ~~assumed~~ considered to be 7.5 hours and a week 37.5 hours unless otherwise stated. When calculating ~~means~~median, if the respondent gave a range of time, then the highest value was used to calculate the mean. Unknown responses were not included in the calculation.

**Formatted:**

Line

spacing:

Double

**Formatted:**

Line

spacing:

Double

### 3.3 Ethics

Whilst the FOI acts allow anyone to submit FOI requests, Ethics~~al~~ approval was obtained for the use of the FOI system ~~to~~ in order to gather research data and the subsequent analysis and publication. (Swansea Medical School Research Ethics Committee (Project reference number 2018-0030).

## 4 Results

### 4.1 Response rate & turnaround time

For the first survey to the universities offering IBMS accredited undergraduate degrees, ~~all~~ 100% (52) responded, while for the second survey to the UK trusts/Health boards with clinical laboratories, 97% (173) responded.

See table 2 for summary of analysis.

~~47~~ Forty seven of the provided Los had multiple outcomes described. ~~42~~ Forty-two of these had 2 outcomes, 1 had 3 and 4 had 4 outcomes described per LO.

We also considered whether the LO action verb appeared on the recommended/avoid list described by Newton~~, Da Silva & Peters~~ et al (29). Verbs on the recommended list appeared in 49% (67/137) LOs ~~75 times~~, whilst ~~42~~ 26% (36/137) of the LOs had an action verb from the avoid list, see appendix 4~~2~~.

**Formatted:**

Keep

with

next

|  | Is the Learning  outcome measurable? | Does the LO describe  POCT  specifically? | Does the LO contain multiple outcomes? | Does the LO contain ~~Is the~~an action verb on the  Recommended list? |
| --- | --- | --- | --- | --- |
| Yes | 84 (61%) | 13 (9%) | 47 (34%) | 67~~75~~ (49%) |
| No | 53 (39%) | 124 (91%) | 90 (66%) | 93~~117~~ (68%) |

Table 2. Analysis of the 137 Point of Care Testing (POCT) learning outcomes (LO) from

Universities providing Institute of Biomedical Science accredited degrees in the United Kingdom.

## ~~Table 4 Point of Care TestingOCT learning outcomes analysis~~

~~The fifth criteria asked, what level of blooms would the action verb be mapped to?~~The level of blooms taxonomy was assessed for each action verb. Of the 54 unique verbs used, 37 could be assigned to a level on blooms taxonomy, 7 were on the avoid list and 9 verbs were not found on any of the lists described by Newton et al (29). One~~1~~ verb, decide, was unmapped as it was split between the Synthesis and Evaluation levels, with 4 lists placing it on the synthesis and 4 on the evaluation, see table 3. For the full list, please see appendix 4~~53~~.

| Witness statement | 67 | 11.3 |
| --- | --- | --- |
| Essay/review | 57 | 9.6 |
| Annotated work | 45 | 7.6 |
| Practical | 28 | 4.7 |
| Audit | 26 | 4.4 |
| Case study | 21 | 3.6 |
| Lecture/tutorial | 20 | 3.4 |
| Personal statement | 6 | 1.0 |
| Self-directed learning | 5 | 0.8 |
| University work | 4 | 0.7 |
| Journal club | 1 | 0.2 |
|  |  | 0.2 |
| MDT (multidisciplinary **team meeting)** | 1 |  |

**Table 4. Types of evidence used by UK clinical laboratories to meet requirements for**

**Health and Care Professions Council (HCPC) standards of Proficiency 14.16 and 14.26**

For the total number of hours spent training on POCT (survey 2, question 3 ~~of the FOI~~), 147 out of the 173~~9~~ laboratories responded. Of these, 81 gave a response for their whole organisation, with the 66 remaining laboratories giving discipline specific responses (i.e., Biochemistry, Microbiology). This resulted in 254 usable data points. Table 5~~46~~ summarizes the responses~~. For~~ ~~full breakdown, please see appendix~~ with appendix 6 providing a full breakdown.

**Formatted:**

Centered

**Formatted:**

Centered

**Formatted:**

Centered

**Formatted:**

Centered

**Formatted:**

Centered

**Formatted:**

Centered

**Formatted:**

Centered

**Formatted:**

Centered

**Formatted:**

Centered

**Formatted:**

Centered

**Formatted:**

Centered

**Formatted:**

Centered

**Formatted:**

Centered,

Keep

with

next

**Formatted:**

Centered

**Formatted:**

Caption,

Left,

Line

spacing:

single

| Useable results | Range of hours | Responses with POCT teaching | Responses with n~~N~~o POCT teaching | Unknown h~~H~~ours POCT teaching | Median~~an~~  (hours) and interquartile  range [Q1-  Q3] |
| --- | --- | --- | --- | --- | --- |

see Appendix 8.

### 5 Discussion

The purpose of this research was to characterize and analyse what, and how much, POCT is taught

~~in 2019 which now includes POCT.~~ accreditation 2020 onwards will have to prove they meet the new QAA statement. This development is bound to affect the amount of POCT taught on these programs.

Of the 253 responses, 19 stated they couldn’t quantify how much time was spent on POCT rotation.

~~154~~

One hundred and fifty

-

four

responses stated that no POCT rotation took place, while a further

26

responded

,

not applicable.

The range of hours on rotation was 0

-

hours. The

450

~~average~~

media

n

(

IQR

)

time spent in a POCT rotation was

0

~~16.9~~

(3.75)

hours

.

For a full breakdown of responses,

at undergraduate

level

and those undertaking the IBMS certificate of competence to enter the

HCPC

register as a

~~biomedical scientis~~

~~t~~

BMS

.

,

prior to the change in QAA benchmark

statement

Both

surveys,

sent

via a

~~freedom of information~~

FOI

request

,

were

sent before the new QAA bench

marking statement for biomedical sciences was released in late 2019, so any university seeking

that only

8

~~13~~

of the universities gave

~~learning outcomes~~

LOs

describing

POCT

specifically

, just

% of the LO

9

s

.

~~A further~~

~~16~~

The remaining

21

universities

gave generic

~~biomedical~~

laboratory

~~learning outcomes~~

LOs

. The

~~rest~~

rem

ainder of universities

,

which includes

u

niversities that stated

~~they did teach POCT and gave teaching hours~~

, provided no

~~learning outcomes~~

LOs

to student

s

.

only do so in a

~~non~~

~~-~~

~~specific way~~

non

-

specific

manner

.

Our

study revealed that only 61% of LO

The first survey is useful to gauge the baseline teaching of POCT at universities. It is worth noting

From this evidence we can infer that the majority of universities that ~~stated they do~~ teach POCT were measurable. Schoepp, in their review of LO in 10 leading universities in 2017, found that only 54% of LO were measurable (22). The verbs were assigned to all 6 levels of the blooms taxonomy, with the LO spread throughout the levels, except evaluation. Scoepp, also mapped the learning outcomes to all levels, however, they found that the middle order, application and analysis, dominated. (22). Thirty-one percent of the LOs used action verbs from the avoid list and 66% contained multiple outcomes, therefore not meeting the described structures for a good LO. It is therefore important that universities review their LO so that future employers will have a clear idea what their trainees/students have been taught. ~~Generic LO are expected, as there will of course~~ ~~be learning that is transferable across all the disciplines used by pathology and perhaps reflects the~~ ~~fact that POCT is not specifically mentioned in the 2015, or its predecessor, QAA benchmark~~ ~~statement.~~ The addition of POCT to the requirements of degree accreditation will require universities to include more POCT specific learning outcomes to their curriculum. We suggest that universities engage with clinical laboratories when writing POCT specific LOs to reflect current practice and refer to both the HCPC standards and QAA benchmark statement for guidance.

Twelve of the universities responded that they had no POCT teaching on their courses and a further nine could not~~n’t~~ give an exact teaching time as POCT was embedded within the curriculum ~~and~~ ~~not taught specifically~~. The median teaching time was 2 hours, and whilst the 2019 QAA benchmark does not stipulate teaching hours, this appears low. The increasing scope of POCT, across the range of disciplines of pathology, merits inclusion across the biomedical science degree curriculum. The LO provided were mostly generic and weren’t reflective of the range of POCT equipment available.

From the university’s perspective, not all graduates

from accredited biomedical science degrees

427

become HCPC registered

~~biomedical scientists~~

BMS

. Therefore, as they only ha

d

to meet the

2015

428

QAA benchmark statement for IBMS degree accreditation, fitting in all topics relevant to

429

~~biomedical scientists~~

BMS

may

not be a priority or fit in with their teaching

program

.

430

~~At this point we need to tie this in with the second~~

~~survey~~

~~to the NHS pathology laboratories~~

The

431

second survey sent to the NHS laboratories assessed trainees/students

undertaking the COC to

432

obtain the core educational requirements as stated by the HCPC

.

433

The training pathway is based on trainees/students undertaking the COC to have the core

434

educational requirements as stated by the HCPC. As the evidence from the first

~~F~~

~~OI request~~

surve

y

435

sent

to the universities showed that a substantial portion of the graduates will not receive much, if

436

any, teaching on POCT then one way to counter this would be

to formalize the POCT

training

of

437

~~those~~

graduates that do

become trainee

~~bio~~

~~medical~~

~~scientists~~

BMS

.

438

The second

survey

to the NHS clinical laboratories asked the question of how many hours are

439

dedicated to POCT teaching.

Forty

-

one

percent

of the returns stated that they

either

had

no POCT

440

training or the POCT teaching was unknown.

This is a surprising finding

,

as all trainee BMS

441

require POCT

teaching/training to meet the HCPC requirements for registration. This

also means

442

that due to

o

no

,

or

often

l

ow

,

teaching h

ours o

f POCT

at

university,

a

large

percentage of newly

443

qualified BMS w

ill not have the skills or knowledge to meet the requirements f

or the

increased

444

use of POCT.

Of the useable returns for this question

, 66 returns

(

%

26

)

stated that they had no

445

~~POCT teaching with a further 37 returns~~

~~(15%)~~

~~stating that the~~

~~(~~

~~POCT)~~

~~teaching~~

~~was unknown.~~

In

446

their national review of English pathology

, Lewis et al (

42)

, the authors looked at

the percentage

447

of

hospital

coagulometer, community glucose and community INR tests were done with

laboratory

448

oversight.

They found that 24%,

65

% and 69% res

pectively

of these tests

were outside of

449

laboratory

oversight

and recommend “

.

*All POCT, wherever it is undertaken, is performed to these*

450

*agreed quality standards, and under a governance structure that is linked to and supported by an*

451

*accredited lab*

”

452

A subsequent question asked what types of

POCT

training was provided in the laboratory for

453

trainees/students.

The two most used training

techniques

w

ere

l

ecture based and practical

454

demonstration

, 35% and 32

respectively.

It’s interesting to note that the t

wo HCPC standards

455

relating to POCT

have different outcomes. Standard 14.16 expects BMS to have knowledge of eth

456

role and responsibilities of the laboratory

with respect to POCT, whilst standard 14.26 expectes

457

BMS to

have a practical

knowledge

of POCT analy

sis

. A combina

tion of the two teaching methods

458

therefore seems appropriate

.

~~3~~

~~9~~

~~% of the training provided used lectures or tutorial style training~~

459

~~and a further 35% of the training was practical based. 10 % stated that they didn’t provide any~~

460

~~training.~~

461

The final question of the second

survey

asked about secondments to a POCT section or department.

462

This question relates to the ‘hands

-

on’ POCT experience that biomedical science trainees

463

experience.

Only 21% of respondents provid

ed

the designated hours for

a secondment

.

Th

erefore,

464

79

% of trainees

will have no practical experience of POCT

in a clinical environment

once

465

qualified

.

~~8~~

~~%~~

~~of respondents~~

~~stat~~

~~ed~~

~~that the hours were unknown, with 6~~

~~1~~

~~% having none, 10%~~

466

~~saying that it wasn’t applicable to their service.~~

This is backed up by the response to question 2

467

asking for the t

ypes of evidence used to meet the

two

HCPC standards

with

POCT

in. 21% of the

468

evidence related to ‘hands on’

experience, Co

mpetency assessments and practical

.

This further

469

**Formatted:**

Font:

Italic

reinforces the possibility of a gap in knowledge in POCT for newly qualified biomedical ~~scientists~~BMS.

The question now becomes - Does the potential gap in POCT teaching and~~&~~ training matter? This

pathology laboratories is limited. This research also revealed ~~shows~~ that in the universities that do ~~teach~~where POCT is taught, it is largely done so in a non-specific or measurable wayit is done so ~~mostly non-specifically, with the majority of LOs provided being generic~~. With the rise in POCT

~~demand~~.

research shows

~~that~~

, in most cases,

the amount of POCT taug

ht at both undergraduate level and

in

being supported by pathology

,

both teaching and training of POCT will have to

b

e formalized, in

order to equip personnel to meet this demand

incre

ase

and become more specific

to meet this

T

his work

provides the

~~creates a~~

baseline for POCT teaching and training in the UK

pr

ior to the

change in QAA benchmark statement

for biomedical science. The challenge now is for universities

providing IBMS

accredited degrees to provide this extra teaching. These universities already have

close links with pathology laboratories as part of their teaching and consideration should be given

qualification in 2019 in POCT, aimed at

~~Biomedical scientists~~

BMS

working, or with an aspiration

~~COVID~~

~~-~~

~~19~~

coro

navirus disease of 2019

pandemic has put a spotlight on POCT due to the use of

this impacts the training and education of

laboratory staff

in POCT will take some time to discover.

to utilizing POCT specialists to support university teaching of POCT.

The research also highlights the lack of specific POCT teaching. The IBMS launched a

to work, in POCT. This was in response to increased calls for a specific qualification (40). The

COVID -19 testing kits and will result in increased exposure to POCT in health systems (41). How The addition of POCT into the QAA benchmark statement and therefore to the IBMS degree accreditation process will impact universities providing IBMS accredited undergraduate degrees. Universities, especially those identified having no POCT teaching, will have to evaluate their curriculum in light of these changes, if they wish to remain accredited. One possible source of increased teaching support will be local p~~P~~athology departments. Whilst the data suggests a lack of POCT training in NHS laboratories for newly qualified ~~Biomedical scientists~~BMS, there are some clear examples where POCT training is provided using a range of training situations and experience.

Whilst this is the first study to review the curriculum for POCT in Laboratory scientists, earlier research in medical (43) and public health curriculums (44) have also reviewed POCT and also found a lack of POCT teaching despite the increased use of POCT in their fields.

**Formatted:**

Line

spacing:

Double

#### 5.1 Limitations

One of the limitations of this study is that the FOI request only went to NHS laboratories. As private businesses are exempt from the FOI act, they were excluded from the study. Eight~~8~~ of the respondents stated that their pathology services were run by private laboratories and so did not provide any data. These 8 responses were counted amongst the unusable returns. Another limitation is that the information provided depends on the respondents interpretation of the request. The FOI acts also allow respondents not to provide the information if it breaches conditions of the acts, such as cost. Two~~2~~ of the respondents used exemptions explicitly and several more stated that they didn’t hold the information in response to individual questions.

5.2 Conclusion

In Summary, it appears that the amount of POCT taught in UK universities is extremely variable and that in the majority of universities it is taught in generic rather than specific terms.

For UK laboratory training in POCT, ~~for trainee BMS’s is variable and~~ the trend is to provide just enough training to meet the HCPC standards, with many laboratories having no rotations in to POCT for practical work experience.

The aim of this study was to get the baseline level of POCT teaching and~~&~~ training for laboratory scientists in the UK. This was done ~~without judgment~~objectively and ~~the information~~ identified~~s~~ a need to further develop POCT teaching and training especially in light of changes in the QAA benchmark statement and increased ~~promotion~~endorsement in government guidelines and reports.

Further ~~work~~ research is needed to see the impact ~~how~~ of both an increased use of POCT and the QAA benchmark changes, in the ~~affect the~~ training and teaching of POCT to students and Trainees in the UK .

**6	Acknowledgements**

**~~6~~7	Conflict of Interest**

None

### ~~7~~8 References

1. Medicines and Healthcare Products Regulatory Agency (MHRA) [In vitro diagnostic point-of-care test devices](https://assets.publishing.service.gov.uk/government/uploads/system/uploads/attachment_data/file/371800/In_vitro_diagnostic_point-of-care_test_devices.pdf) (2021) available at [https://www.gov.uk/government/publications/in-vitro-diagnostic-point-of-care-testdevices](https://www.gov.uk/government/publications/in-vitro-diagnostic-point-of-care-test-devices) [Cited June 29, 2021].
2. Vashist SK. Point-of-Care Diagnostics: Recent Advances and Trends. Biosensors.

2017;7(4). <https://doi.org/10.3390/bios7040062>

3. Park HD. Current Status of Clinical Application of Point-of-Care Testing. Archives of pathology & laboratory medicine. 2021;145(2):168-75.

<https://doi.org/10.5858/arpa.2020-0112-RA>

4. Welsh Government, Healthcare Science in NHS Wales, Looking forward. Available at [https://gov.wales/sites/default/files/publications/2019-03/healthcare-science-in-nhswales.pdf](https://gov.wales/sites/default/files/publications/2019-03/healthcare-science-in-nhs-wales.pdf) [Cited July 2nd, 2021].
5. Welsh Government, Pathology Statement of Intent (2019) available at <https://gov.wales/written-statement-precision-medicine> [Cited July 2nd, 2021].
6. Scottish government, Driving improvement, delivering results: healthcare science national delivery plan 2015-2020 (2015) [https://www.gov.scot/publications/](https://www.gov.scot/publications/driving-improvement-delivering-results-scottish-healthcare-science-national-delivery-plan-20152020/) [Cited July

2nd, 2021].

7. NHS England, NHS Long term plan (2019) available at <https://www.longtermplan.nhs.uk/publication/nhs-long-term-plan/> [Cited July 2nd, 2021].
8. NHS England, Science in Healthcare: Delivering the NHS long term plan – The Chief scientific Officer’s strategy (2020) available at [https://www.england.nhs.uk/](https://www.england.nhs.uk/wp-content/uploads/2020/03/science-in-healthcare-delivering-the-nhs-long-term-plan.pdf) [Cited July

2nd, 2021].

9. Institute of Biomedical Sciences (IBMS) available athttps://www.ibms.org/ [Cited June 29, 2021].
10. Health and Care Professions Council (HCPC) Standards of proficiency for biomedical scientists (2014) available at [https://www.hcpc-uk.org/standards/](https://www.hcpc-uk.org/standards/standards-of-proficiency/biomedical-scientists/) [Cited June 29, 2021].
11. Quality Assurance Agency (QAA) Subject Benchmark Statement, biomedical sciences (2019) available at [https://www.qaa.ac.uk/](https://www.qaa.ac.uk/docs/qaa/subject-benchmark-statements/subject-benchmark-statement-biomedical-sciences.pdf?sfvrsn=2bf2c881_4) [Cited June 29, 2021].
12. Institute of Biomedical Science (IBMS) Criteria and Requirements for the Accreditation and Re-accreditation of BSc (Hons) in Biomedical Science (2017) available at [https://www.ibms.org/resources/](https://www.ibms.org/resources/documents/criteria-and-requirements-for-the-accreditation-and-re/) [Cited June 29, 2021].
13. Quality Assurance Agency (QAA), Subject Benchmark Statement, biomedical sciences (2015) available at [https://www.ibms.org/](https://www.ibms.org/resources/documents/qaa-subject-benchmark-statement-biomedical-sciences/) [Cited July 2nd, 2021].
14. Gosling D, Moon J. How to use learning outcomes and assessment criteria. London:

SEEC; 2002.

15. Jenkins A, Unwin, D.. How to write learning outcomes. 2001. Available from:

<https://www.ubalt.edu/> [Cited July 2nd, 2021].

16. Adam S. A consideration of the nature, role, application and implications for European education of employing ‘learning outcomes’ at the local, national and international levels. University of Westminster. 2004 Jun.
17. Moon J. The module and programme development handbook: A practical guide to linking levels, outcomes and assessment criteria. Routledge; 2003 Dec 16.
18. Harden~~ARDEN~~ RM. AMEE Guide No. 14: Outcome-based education: Part 1-An

introduction to outcome-based education. Medical teacher. 1999;21(1):7–14.

19. Kennedy D, Hyland Á, Ryan N. Writing and Using Learning Outcomes: A Practical Guide. 2007. Available at [https://www.fibaa.org/](https://www.fibaa.org/fileadmin/uploads/content_uploads/Writing_and_Using_Learning_Outcomes_01.pdf#:~:text=Implementing%20Bologna%20in%20your%20institution%20C%203.4-1%20Using,as%20the%20QAA%20%28Quality%20Assurance%20Agency%20for%20Higher) [cited July 2nd, 2021].
20. Mahajan M, Singh MK. Importance and benefits of learning outcomes. IOSR Journal of Humanities and Social Science. 2017;22(03):65-7.
21. Prideaux D. The emperor's new clothes: from objectives to outcomes. Medical education. 2000;34(3):168-9.
22. Schoepp K. The state of course learning outcomes at leading universities. Studies in Higher Education. 2019;44(4):615-27.
23. Chatterjee D, Corral J. How to write well-defined learning objectives. The journal of education in perioperative medicine: JEPM. 2017 Oct;19(4).
24. Carr JW, Hardin S. The key to effective assessment: Writing measurable student learning outcomes. Recreational Sports Journal. 2010 Oct;34(2):138-44.
25. Wittmann-Price RA, Fasolka BJ. Objectives and outcomes: The fundamental difference.

Nursing education perspectives. 2010 Jul 1;31(4):233-6.

26. Bloom BS. Taxonomy of educational objectives: The classification of educational goals. Cognitive domain. 1956.
27. Krathwohl DR. A revision of Bloom's taxonomy: An overview. Theory into practice. 2002 Nov 1;41(4):212-8.
28. Stanny CJ. Reevaluating Bloom’s Taxonomy: What measurable verbs can and cannot say about student learning. Education Sciences. 2016 Dec;6(4):37.
29. Newton PM, Da Silva A, Peters LG. A Pragmatic Master List of Action Verbs for Bloom's

Taxonomy. Frontiers in Education. 2020;5(107).

30. UK government, How to make a freedom of information (FOI) request available at <https://www.gov.uk/make-a-freedom-of-information-request> [Cited July 2nd, 2021].
31. Fowler AJ, Agha RA, Camm CF, Littlejohns P. The UK Freedom of Information Act (2000) in healthcare research: a systematic review. BMJ Open. 2013;3(11):e002967– e002967.
32. Knight J, Stead AP, Geyton TO. Comparing the academic performance of graduate-entry and undergraduate medical students at a UK medical school. Education for health (Abingdon, England). 2017;30(1):75–8.
33. Phillips AW, Friedman BT, Utrankar A, Ta AQ, Reddy ST, Durning SJ. Surveys of health professions trainees: prevalence, response rates, and predictive factors to guide researchers. Academic Medicine. 2017 Feb 1;92(2):222-8.
34. Arulrajah P, Steele S. UK medical education on human trafficking: assessing uptake of the opportunity to shape awareness, safeguarding and referral in the curriculum. BMC medical education. 2018 Dec;18(1):1-7.
35. Ayton A, Ibrahim A. Does UK medical education provide doctors with sufficient skills and knowledge to manage patients with eating disorders safely?. Postgraduate medical journal.

2018 Jul 1;94(1113):374-80.

36. Copeland C, Barron DT. "Delirium: An essential component in undergraduate training?". Nurse education today. 2020;85:104211.
37. Ormerod R. The history and ideas of pragmatism. Journal of the Operational Research Society. 2006 Aug;57(8):892-909.
38. Duram L. Pragmatic Study. Vol. 2, Encyclopedia of Research Design. 2010. p. 1072–4.

636

637

| **What is the scope of teaching and training of undergraduate students and trainees in Point of Care Testing in UK universities and hospital laboratories?** | | | | |  | |
| --- | --- | --- | --- | --- | --- | --- |
| *Learning outcome: a statement of what a learner is expected to know, understand and/or be able to demonstrate at the end of a period of learning”.* | | Gosling and Moon, 2001  (14) | |  |  |  |
| *A learning outcome is a written statement of what the successful student/learner is expected to be able to do at the end of the module/course unit or qualification.* | | Adam, 2004 (16) | |  |  |  |
| *A learning outcome is a statement of what a learner is expected to know, understand and be able to do at the end of a period of learning and of how that learning is to be demonstrated”.* | | Moon, 2002 (17) | |  |  |  |
| *In outcome-based education, the outcomes agreed for in the curriculum guide what is taught and what is assessed.* | | Harden, Crosby & Davis  1999 (18) | |  |  |  |
| *Learning outcomes are statements of what a learner is expected to know, understand and/or be able to demonstrate after completion of a process of learning* | | Kennedy, Hyland & Davis  2007 (19) | |  |  |  |
| Appendix 2 ~~Table 2~~ Suggested structures of course learning outcomes. | | | |  |  |  |
| Structure | Reference | | Good LO example |  |  |  |
| Begin with a consistent short stem: such as “Students will be able to” or “At course completion students “ | Schoepp  2017 (22) | | Students can critically | re | view |  |
|  |  |  | the methodology of a r | es | earch |  |
|  |  |  |  |  |  |  |
| 37 | | | | |  |  |

638

State the desired student performance/behavior using

concrete action, or operational, verbs such as

create,

apply, interpret, describe, identify, categorize

eting

study published in a mark

journal.

SMART objectives

**S**

pecific: What action will be performed and by whom?

**M**

easurabl

measured?

e:

How

will

success

be

Objectives should quantify the amount of change

expected.

**A**

chievable: Can this objective be achieved within a

given time frame and with available resources?

**R**

elevant: Are the objectives aligned with the

instructional metho

d and assessment?

**T**

ime

-

Bound: When will this objective be achieved?

Objectives should provide a time frame indicating when

the objective will be met.

Chatterjee

and Corral

2017 (23)

Upon

completion

of

the

difficult

airway

works

hop,

participants should b

e ab

le to

formulate an accurate algor

ithm

for the management of an o

bese

adult patient with inade

quate

ventila

mask

face

tion,

according to the Ame

rican

gists

Society of Anesthesiolo

difficult airway algorithm

The ABCD method

A = Audience is the target

group of the LO. The

learning outcomes statement would begin something

like: “The students will . . .” or “The participants will .

. .”

and

Carr

Hardin

2010 (24)

In response to four scena

rios,

will

each student staff member

ps

correctly articulate the ste

of

Plan

the Emergency Action

procedures as outlined in

the

student staff manual.

formulate	1 Highlight	1 execute	1 undertake	1 appraise	1 benchmark	1 outline	1 conduct	1 plan	1 contribute	1 provide	1 criticise	1 receive	1 design	1 record	1 arrange	1 relate	1 consider	1 review	1 decide	1 satisfy	1 compare	1 select	1 differentiate	1 summarise	1 cost	1 theoretically underpin	1 appreciate	2 uphold	2 reflect	2 know	2 comply	2 identify	2 respect	2 acquire	3 compose	3 justify	3 use	3 aware	4 communicate	4 work safely	4 apply	4 perform	5 develop	6

know	7

recognise

7

analyse

8

describe

9

interpret

10

discuss

10

demonstrate

11

explain

12

evaluate

13

understand

24

**Grand Total**

**192**

641

Appendix

4

~~2~~

642

**~~Row Labels~~**

**Count of Avoid lis**

**t**

~~ARRANGE~~

~~-~~

~~AVOID~~

~~1~~

~~SELECT~~

~~-~~

~~AVOID~~

~~1~~

~~RELATE~~

~~-~~

~~AVOID~~

~~1~~

~~APPRECIATE~~

~~-~~

~~AVOID~~

~~2~~

~~AWARE~~

~~-~~

~~AVOID~~

~~5~~

~~KNOW~~

~~-~~

~~AVOID~~

~~9~~

~~UNDERSTAND~~

~~-~~

~~VOID~~

~~23~~

**~~Grand Total~~**

**~~42~~**

643

~~Appendix~~

~~5~~

~~3~~

count of action verb by level of blooms taxonomy

644

**Row Labels**

**Count of Masterlist (time on source lists)**

**Analysis**

**12**

Analyse

8

Appraise

1

Compare

1

Differentiate

1

Outline

1

**Application**

**33**

Apply

4

Conduct

1

Contribute

1

Criticise

1

Demonstrate

11

Execute

1

Interpret

10

Provide

1

Use

3

**Avoid	42**

| Appreciate | 2 |
| --- | --- |
| ARRANGE | 1 |
| Aware | 5 |
| know | 9 |
| RELATE | 1 |
| SELECT | 1 |
| Understand | 23 |
| **Comprehension** | **30** |
| Discuss | 10 |
| Explain | 12 |
| Explore | 1 |
| Perform | 5 |
| Review | 1 |
| Summarise | 1 |
| **Evaluation** | **22** |
| Develop | 6 |
| Evaluate | 13 |
| Justify | 3 |
| **knowledge** | **23** |
| Acquire | 3 |
| Describe | 9 |
| highlight | 1 |
| Identify | 2 |
| Recognise | 7 |
| Record | 1 |
| **not on list** | **14** |
| benchmark | 1 |
| Cost | 1 |
| receive | 1 |
| Respect | 2 |
| satisfy | 1 |
| theoretically underpin | 1 |
| undertake | 1 |
| uphold | 2 |
| work safely | 4 |
| **Synthesis** | **15** |

Communicate	4

Comply	2

Compose	3

Consider	1

Design	1

Formulate	1 Plan	1

|  |  |  | **teaching**  **(n)** |  |  |
| --- | --- | --- | --- | --- | --- |
| whole trust response | 81 | 21 | 13 | 0-150 | 17.6 |
| Specialist laboratories | 7 | 0 | 2 | 1-7 | 3.4 |
| Histology/cytology | 39 | 19 | 4 | 0-15 | 2.7 |
| Immunology | 7 | 4 | 2 | 0-7 | 1.4 |
| Haematology | 29 | 4 | 6 | 0-37.5 | 6.6 |
| Biochemistry | 24 | 2 | 2 | 0-450 | 76.2 |
| Blood sciences | 21 | 1 | 1 | 0-112.5 | 17 |
| Microbiology/virology | 46 | 15 | 7 | 0-15 | 2.3 |

650

651	Appendix 7~~75~~ response to question 4 regarding training methods for POCT in clinical 652	laboratories.

| **Laboratory** | **Useable responses** | **No training** | **Practical training** | **lecture/ tutorial** | **shadowing**  **/ tour** | **Selfdirected** | **E-learning** | **University** |
| --- | --- | --- | --- | --- | --- | --- | --- | --- |
| Whole trust  response | 77 | 12 | 42 | 44 | 21 | 3 | 4 | 5 |
| Specialist laboratories | 7 | 0 | 6 | 5 | 3 | 1 | 0 | 1 |
| Histology/ cytology | 27 | 6 | 9 | 14 | 8 | 3 | 1 | 0 |
| Immunology | 5 | 3 | 2 | 2 | 0 | 0 | 0 | 0 |
| Haematology | 27 | 4 | 12 | 16 | 6 | 2 | 1 | 0 |
| Biochemistry | 23 | 1 | 20 | 13 | 5 | 0 | 2 | 0 |
| Blood sciences | 21 | 1 | 14 | 14 | 9 | 1 | 0 | 0 |
| Microbiology/ virology | 38 | 7 | 14 | 22 | 9 | 3 | 1 | 0 |

653

| Appendix 8~~86~~, response to question 5 regarding rotations into a POCT section/department. | | | | | | |
| --- | --- | --- | --- | --- | --- | --- |
| **Laboratory** | **Useable results** | **Range of hours** | **No**  **POCT**  **rotation** | **N/A** | **Unknown** | **Mean** |
| whole trust response | 90 | 0-450 | 56 | 7 | 10 | 18.2 |
| Specialist laboratories | 12 | 0-7.5 | 7 | 4 | 0 | 0.94 |
| Histology/cytology | 38 | 0-3.75 | 28 | 7 | 0 | 0.73 |
| Immunology | 8 | 0-7.5 | 5 | 2 | 0 | 1.25 |
| Haematology | 26 | 0-30 | 22 | 0 | 0 | 2.2 |
| Biochemistry | 23 | 0-450 | 7 | 0 | 2 | 86.7 |
| Blood sciences | 16 | 0-112.5 | 6 | 0 | 3 | 17.3 |
| Microbiology/virology | 40 | 0-15 | 23 | 6 | 4 | 1.6 |

654

655

Response to editor and reviewers

RE: PONE-D-21-35377

Dear Editor,

Many thanks to both you and the reviewers for taking the time to review our manuscript and providing welcome feedback and comments. See below our response to each point raised. The authors have carefully considered the comments and tried our best to address every one of them. We hope the manuscript after careful revisions meet your standards. The authors welcome further constructive comments, if any.

Minor changes:

Authors details:

1. Line 1: Philip M. Newton. Provide the full middle name **Response: Middle name provided**

Discussion:

1.) Line 339. Provide recommendations on possible LO that could be used to standardised POCT. **Response: Added a paragraph suggesting liaising with laboratory colleagues and referencing the HCPC and QAA in revised LO**

Major changes:

Abstract:

1.) Line 14. Write out in full United Kingdom (UK)
2.) Line 16. Lowercase **freedom of information**
3.) Line 17, add a comma after **teaching of POCT,**
4.) Line 21. Change 12 to **Twelve**
5.) Line 22. Change 66 to **Sixty-six**
6.) Line 22. 66 laboratories replied they had no POCT training. Change to:

**Sixty-six laboratories confirmed that there was no POCT training.**

7.) Line 23. Provide a median for the university and laboratory hours. Confirm if the range of teaching hours was 0-35 or 1-35.
8.) Line 29-32. Break into two separate sentences
9.) Line 26. 31% Is not mentioned in the results. Is this the 42 out of 117? Please add percentages to Table 4 column 5.

**Response: All revised accordingly.**

Introduction:

The introduction is too long ~10 pages versus the combined methods, results, discussion ~10.

This needs to be shortened to include only relevant information.

1.) Line 35. Write out United Kingdom (UK)
2.) Line 35. Products is lower case products
3.) Line 38. near patient testing, use lower case
4.) Line 39-43. Provide examples of commonly used POCT, and comment on the impact of COVID testing.
5.) Line 45. Change strategy to strategies
6.) Line 45 at a comma after An example is, 7.) Line 52, remove italics for will 8.) Line 65.

Fig 1 The 4 routes to Health and Care Professions Council registration for biomedical scientists (adapted from information from IBMS on becoming HCPC registered (9)).

Change to:

Fig 1. The four routes to Health and Care Professions Council registration for biomedical scientists (adapted from information from IBMS on becoming HCPC registered (9)).

Write out the full name for the abbreviations COC, IBMS, HCPC, BMS in a footnote. Provide more explanation in the figure for what is meant by top up education and equivalence process.

**Response: All revised accordingly.**

2.1

1.) Line 59-74 needs to be shortened and summarised (points to include)
    - Biomedical scientists are registered with the HCPC
    - What defines a biomedical scientist in terms of qualification
    - Briefly describe the routes to qualification described in your figure 1 -	What is a COC

**Response The portion of the manuscript has been shortened to only include the suggested relevant points.**

2.) IBMS. Write out full name when abbreviating for the first time
3.) Line 61 Biomedical scientists is given an abbreviation but this is not used consistently throughout the paper, please abbreviate all subsequent biomedical scientist to BMS

**Response: All revised, as suggested, except line 65. Biomedical scientist hasn’t been abbreviated as BMS in this instance, as “BMS” is not the protected title, “Biomedical scientist” is.**

2.2

1.) Line 76-111 needs to be shortened and summarised (points to include) -	What defines an accredited biomedical degree
    - The function of the QAA benchmarking for biomedical degrees
    - What QAA don’t provide i.e. national curriculum, only general guidelines
    - HCPC references to POCT
    - Addition of POCT to the 2019 QA benchmark statement (As compared with 2015 statement) **Response: The portion of the manuscript has been shortened to only include the suggested relevant points.**

2.) Line 95-107. Use abbreviation **NPT**

**Response: revised accordingly**

2.3:

1.) Line 113-154 requires significant shortening (points to include) - Include: Gosling and Moon define a learning objective as….

- There is no standard definition of a LO. Place table 1 as an appendix, and make reference to it here.
- What is the purpose of a LO
- What defines a good learning objective. Place Table 2 as an appendix, and refer to it here. - Bloom’s Taxonomy: short comment on what it is and what it is used for.

**Response: The portion of the manuscript has been shortened to only include the suggested relevant points.**

2.4:

1.) Line 156-181: needs to be shortened and summarised (points to include)

- What is a FOI request and governed by which acts
- Who can you request this from (public sector)
- How do you request (in writing)
- Higher response rates when made via a FOI request

**Response: The portion of the manuscript has been shortened to only include the suggested relevant points.**

2.) Line 156-157: Inconsistency in capitals and lower case, freedom of information act. Please correct

**Response: revised accordingly**

3.) Line 173-176 – this is describing the method of the study (remove from the introduction)

**Response: Removed**

4.) Line 82-198: needs to be shortened and summarised to include only the aims of the research
    - How much (dedicated time) POCT teaching and training is there at universities and NHS laboratories
    - How is POCT taught

**Response: The portion of the manuscript has been shortened to only include the suggested relevant points.**

Methods:

1.) Line 199. Change heading to Materials and Methods 2.) Line 201. Grammar. Change to:

May 2018, with a second survey sent to all the NHS trusts/Health board laboratories in the UK in

3.) Line 202-203: Table 3 gives the wording of each of the surveys sent via freedom of information requests. Change to,

The wording for each survey is provided in Table 3

4.) Table 3: Survey 1: Point 3: correct the spelling of leaning to learning 5.) Lines 204-205

Insert the table title as a label using the caption option (above the table)

Reword the title to indicate who was assessed in survey 1 (Universities) and 2 (NHS Laboratories). Change to:

Table 3. The survey questions which were used to assess teaching and training of Point of Care Testing at universities (survey 1) and NHS laboratories (survey 2).

Provide the full name and abbreviation for IBMS, HCPC in a footnote or write it out in full in the table.

6.) Reword line 206-207. The questions used in the first survey seek to gather information on how much POCT is taught and the POCT topics covered at the selected universities.

The questions used in the first survey seek to gather information on the time allocated to POCT teaching at the selected universities and the topics covered.

**Response: revised accordingly**

7.) Reword lines 209-214.

As described in the introduction, this information will cover trainees/students going through pathways 1-3 to HCPC registration. The first survey will cover pathways 1 and 2 as students /trainees going down these pathways will have completed an accredited BMS degree, whilst the second survey covers 1-3 as all students/trainees going through these pathways will have to have a clinical laboratory placement and achieve the COC.

The separate surveys were done in order to assess the different avenues of HCPC registration of trainees/ students (refer to Fig 1). The participants in survey 1 complete an accredited BMS degree (pathways 1 and 2) whilst those of survey 2 achieve a COC during a clinical laboratory placement.

**Response: Passage reworded as suggested.**

Selection process:

1.) Line 225, 227. Please clarify how the NHS acute trust/ Health board/ Blood transfusion trusts would assist in locating pathology laboratories. Are these institutions where training Biomedical scientist are registered / perform part of their training ?

**Response: Added line explaining that these locations are where pathology laboratories are located.**

2.) Reword 228-229. This resulted in 179 FOI requests. The FOI requests were sent to each university/Trust/Health board dependent on the instructions on their internet site.

This identified 179 institutions (university/ trust/ health boards) where FOI requests could be sent.

**Response: Reworded, as suggested.**

Data analysis:

1.) Line 233. For the hours tested, the data is likely to be skewed. Check if the data is normally distributed ? (use a mean) or skewed ? (use a median).
2.) Line 233-234. Place range, median, IQR within brackets.

Response rate, response turnaround time, teaching and training hours (range, median, interquartile range) were calculated.

3.) Line 235. Add (LO) after learning objective
4.) Line 239. Add et al.

Is the action verb used on the recommended/avoid list as described by Newton et al.(29)?

5.) Line 241. Please clarify in the text, did one author assess and one author review or did all review?
6.) Line 241-242. Any changes were agreed by all 3 authors. Change to:

Any changes required agreement by all three authors.

7.) Line 242-243. Rather use et. al

Supplementary data from Newton et al.(29) was used.

8.) Line 244. It is unclear how the verbs were assigned. Was it based on the level provided by the authors (29), what is meant by ‘appeared in the majority of the lists searched’. Please reword.
9.) Line 245. Change assumed to considered
10.) Line 246, 247. If using median (based on distribution of your data), change mean to median
11.) Line 249. Change Ethical to ethics.
12.) Adjust grammar 249-251

Whilst the FOI acts allow anyone to submit FOI requests, ethics approval was obtained for the use of the FOI system in order to gather research data and the subsequent analysis and publication (Swansea Medical School Research Ethics Committee (Project reference number 2018-0030)).

**Response: revised accordingly**

Results:

4.1:

1.) Line 254. Remove all.

For the first survey to the universities offering IBMS accredited undergraduate degrees, 100%

**Response: Removed**

4.2**:**

1.) Line 260-262. Line 261 remove accredited (repeated), reworded:

Fifty-two universities responded and gave results for 55 accredited degree programs. Of these, 9 (16%) described their POCT teaching as embedded within the curriculum and so teaching hours could not be quantitated.

2.) Line 264. Need to provide a median (IQR) of hours.

The amount of direct teaching, non-embedded, received by an undergraduate on POCT ranged from 1 to 35 hours with a median (IQR) of….

3.) Does the amount of teaching not range from 0 to 35 hours?
4.) Line 266-267

Write out POCT, IBMS in full, in the title. Need to stipulate whether median hours.

5.) Line 268, 269, 273. Use LO abbreviation.
6.) Line 268: Change (FOI question 3) to (survey 1, question 3)
7.) Line 270-272. Reword to:

There were 192 action verbs, of which 54 were unique (see Appendix 1).

8.) Line 273, change 13 to Thirteen
9.) Line 275, change 47 to Forty-seven change 42 to Forty-two
10.) Line 278, change Newton, Da Silva & Peters to Newton et al.(29)
11.) Line 278-279, please clarify, the measure for the recommended list verbs is number of appearances (? Number in total or number of LO containing recommended verbs), whilst the measure for the avoid list verbs is number of LO with the verb.
12.) Table 4 is not referenced to in the text. Please add. Provide percentage in brackets for numbers in columns 2-5

Insert a line for total

?117 correct in the table ? 42

Write out POCT in full in the caption and define LO in the caption. Use Table caption function

13.) Line 281. Change: The fifth criteria asked, what level of blooms would the action verb be mapped to? to :

The level of blooms taxonomy was assessed for each action verb.

14.) Refer to Table 5 in the text.
15.) Line 283. Add Newton et al.

lists described by Newton et al.(29). Change 1 to One

16.) Line 287-288.

Insert title label using table caption option Change to:

Table 5. Action verbs mapped to levels of Bloom’s taxonomy. The verbs were assigned using the lists collated by Newton et al.(29)

**Response: revised accordingly**

4.3:

1.) Line 289 change 4.3 POCT training in NHS to

4.3 POCT training in NHS laboratories

2.) Line 290.

Change (question 3 of the FOI) to

(survey 2, question 3)

3.) Line 290. In line 256 it states that 173 laboratories responded whilst in line 290 it states 179. Please clarify.
4.) Line 292. Change remaining laboratories giving discipline specific responses (i.e., Biochemistry, Microbiology) This resulted in 254 usable data points. Table 6 summarizes the responses. For full breakdown, please see appendix to:

remaining laboratories giving discipline specific responses (i.e. biochemistry, microbiology). This resulted in 254 usable data points. Table 6 summarizes the responses with appendix 4 providing a full breakdown.

Table 6, use the caption insert option. Change title to

Table 6. Summary of hours dedicated to teaching of Point of Care Testing in the NHS.

Change to mean to 1

Write out POCT or provide abbreviation in caption.

Lower case No and Hours in the table

5.) Line 296-301

Line 299-301 should come before line 296-298 Line 296. Provide the median (IQR) Reword to:

Of the 254 responses, 37 stated that they could not quantify the POCT training with a further 66 reporting no POCT training. In the remaining 151, a range of 0-450 hours was dedicated to POCT training. Overall, the median (IQR) hours for teaching of POCT was …. for NHS clinical laboratories. In cases where a range of hours was provided by a respondent, the maximum value was used for consistency.

6.) Line 302 to 304. ? appendix 5 Reword to:

Fig 3, shows the different training methods used in laboratory teaching of POCT, provided in response to question 4 of survey 2. For a breakdown between the whole trust responses and the different pathology disciplines see appendix 5.

7.) Line 305-306.

Reword caption:

Fig 3. The different training methods used by UK NHS laboratories for teaching and training of Point of Care Testing in 2018.

8.) Line 307-310. As you can see in fig 3, practical training and lecturebased training was the most popular training method Reword to:

As seen in Fig 3, practical training and lecture based training were the most popular training methods

Unclear on the statistics. 225 usable responses versus the 372 total. Please clarify in your text.

9.) Line 309, change 34 to Thirty-four
10.) Table 7. Provide % for columns 3-6 Insert title label using caption function Write out LO and POCT in full in the table Rename caption:

Table 7. Summary of time spent on Point of Care Testing rotation in UK NHS laboratories.

Provide a median (IQR)

11.) Line 311-315

Question 5 asked if there was any rotation into the POCT section or department for the trainee/student.

Table 7 shows the range of POCT rotations received in the response. 134 laboratories responded to this question with 90 responding for the whole trust. The remaining 163 gave discipline specific information, resulting in 253 useable responses

Reword to:

Question 5 asked if there was any rotation into the POCT section or department for the trainee/student. One hundred and thirty-four laboratories responded to this question with 90 responding for the whole trust. The remaining 163 gave discipline specific information, resulting in 253 useable responses (see Table 7).

12.) Line 317-320

Of the 253 responses, 19 stated they couldn’t quantify how much time was spent on POCT rotation. 154 responses stated that no POCT rotation took place, while a further 26 responded not applicable. The range of hours on rotation was 0-450 hours. The average time spent in a POCT rotation was16.9 hours

Reword:

Of the 253 responses, 19 stated they could not quantify how much time was spent on POCT rotation. One hundred and fifty-four responses stated that no POCT rotation took place, while a further 26 responded, not applicable. The range of hours on rotation was 0-450 hours. The median (IQR) time spent in a POCT rotation was … hours.

Provide a median (IQR)

**Response: revised accordingly**

13.) What results did you obtain for question 2 of survey 2, types of evidence. Please address in your results.

**Response: Apologies, unsure how this was missed. Results for question 2 added and discussed.**

**Discussion:**

1.) Line 322. The research was also intended to determine how much time is dedicated to POCT teaching. Please add this.
2.) Line 322-325. Separate into two separate sentences (too long).
3.) Line 324 and 326 are repetitive about the change in the QAA benchmark statement. Combine these two.
4.) Line 326. Use the FOI abbreviation
5.) Line 330. Add at universities after POCT.

The first survey is useful to gauge the baseline teaching of POCT at universities.

6.) Line 331, 332, 333, 339 use LO abbreviation
7.) Line 331. I cannot find reference of the 16 generic LO in the results?

**Response: revised accordingly**

8.) Line 332-333. Reword. The rest, which includes universities that stated they did teach POCT and gave teaching hours, provided no learning outcomes.

The remainder of universities where hours were allocated to teaching of POCT, provided no LO to students.

**Response: Reworded to:**

**“The remainder of universities provided no LO to students.”**

**Not all of the remaining universities provided hours, although some of them did.**

9.) Line 333-335. Reword

From this evidence we can infer that the majority of universities that stated they do teach POCT only do so in a non-specific way.

From this evidence we can infer that the majority of universities that teach POCT do so in a nonstandardised manner.

**Response: Reworded to**

**“From this evidence we can infer that the majority of universities that teach POCT do so in a non-specific manner. “**

**We kept non-specific as the LO weren’t specific to POCT and were generic in nature**

10.) Line 335-337 it is unclear what point you wish to make, please reword.

Generic LO are expected, as there will of course be learning that is transferable across all the disciplines used by pathology and perhaps reflects the fact that POCT is not specifically mentioned in the 2015, or its predecessor, QAA benchmark statement.

**Response: Removed from manuscript**

11.) Line 341. Change couldn’t to could not
12.) Line 341-342. Remove ‘and not taught specifically’

**Response: revised accordingly**

13.) Line 340-345. Provide a comment on results you obtained for median hours taught/ trained. Do you feel that it is an adequate amount of time, what would be your recommendation? How

many hours does the current QAA benchmark stipulate and elaborate on the requirements for POCT teaching and training currently in 2021?

**Response: Added comment of teaching hours and suggested increase in time to reflect growing scope.**

14.) Line 346 to 348.

At this point we need to tie this in with the second survey to the NHS pathology laboratories. The training pathway is based on trainees/students undertaking the COC to have the core educational requirements as stated by the HCPC.

Reword:

The second survey sent to the NHS pathology laboratories assessed trainees/ students undertaking the COC to obtain the core educational requirements as stated by the HCPC.

15.) Line 348.

As the evidence from the first FOI request to the universities showed that a substantial portion of the graduates will not receive much, if any, teaching on POCT then one way to counter this would be training those graduates that do become trainee biomedical scientists. Change to : (please check if this is the point you were wanting to make)

As the evidence from the first survey sent to the universities showed that a substantial portion of the graduates will not receive much, if any, teaching on POCT, then one way to counter this would be to formalise the POCT training of graduates that become trainee biomedical scientists.

**Response: revised accordingly**

16.) Line 352-363. This is a repetition of the results section. Instead of restating the results, speak to the 41% (26+15) that have no formal POCT training. Does this meet your suggestion of training trainee biomedical scientists (line 350-351)? Tie these thoughts together

Regarding type / method of training, don’t restate results, comment that lectures and practical were the most used. Does didactic teaching work or do you recommend a combination? Hands on experience, again summarise the main points. 79% had no hands on experience? What is the value of hands on experience?

**Response: Added comment to address points raised.**

17.) Line 364. Change & to and
18.) Line 365. Grammar research shows that, in most cases, the amount of POCT taught at both undergraduate level and in change to

research shows, that in most cases, the amount of POCT taught at both undergraduate level and

in

**Response: revised accordingly**

19.) Line 366-367

This research also shows that in the universities that do teach POCT it is done so mostly nonspecifically, with the majority of LOs provided being generic.

Change to : (please check if this is the point you were wanting to make)

This research also revealed that in universities where POCT is taught, it is largely done so in a non-standardised or measurable way.

**Response: Reworded to**

**“This research also revealed that in universities where POCT is taught, it is largely done so in a non-specific or measurable way. “**

**Universities provided LO that weren’t specific to POCT, instead provided generic LO**

20.) Line 367-369:

With the rise in POCT being supported by pathology both teaching and training of POCT will have to increase and become more specific to meet this demand.

Change to: (please check if this is the point you were wanting to make)

With the rise in POCT being supported by the pathology discipline, both teaching and training of POCT will have to be formalised, in order to equip personnel to meet this demand.

21.) Line 370

This work creates a baseline for POCT teaching and training in the UK prior to the change in QAA Change to:

This research provides the baseline of POCT teaching and training in the UK prior to the change in QAA

22.) Line 374. Change specialist to specialists
23.) Line 377. Add the work specific after increased calls for a
24.) Line 377. Write out COVID-19. coronavirus disease of 2019
25.) Line 384. Add a comma after changes
26.) Line 385. Lowercase Pathology to pathology

**Response: revised accordingly**

27.) Please address your results of the action verbs, taxonomy. Why is it important to use these verbs in future LO.

**Response: Added comments in discussion to address action verb use.**

5.1 Limitations:

1.) Line 390. Change 8 to **Eight**
2.) Line 395: Change 2 to **Two**

**Response: revised accordingly**

Summary:

1.) Line 397. Add Heading: Conclusion
2.) Line 398. Add it after universities
3.) Line 399-400. The concept of variable training at universities has been repeated here.

The sentence starts with universities and then talks about laboratories. Please reword.

4.) Line 401. Change & to and
5.) Line 402-404.

This was done without judgment and the information identifies a need to further develop POCT teaching and training especially in light of changes in the QAA benchmark statement and increased promotion in government guidelines and reports.

Change to : (please check if this is the point you were wanting to make)

This was done objectively and identified a need to further develop POCT teaching and training, especially in light of the changes in the QAA benchmark statement and increased endorsement in government guidelines and reports.

6.) Line 405-406

Further work is needed to see how both increased use of POCT and the QAA benchmark changes affect the training and teaching of POCT to students and Trainees in the UK .

Change to

Further research is needed to see the impact of both an increased use of POCT and the QAA benchmark changes, in the training and teaching of POCT to students and trainees in the UK.

7.) Line 407. Add an Acknowledgments section

**Response: revised accordingly**

References:

1.) Line 431, 433, 438, 499, 501. Put a space after org/
2.) Line 453, Author name is in capitals, please correct
3.) Line 504, add a Heading Supporting information

**Response: revised accordingly**

General journal guidelines:

1.) Make sure the entire text is double-spaced

**All text is now double-spaced**

Once again, thank you for taking the time for reviewing our manuscript and hopefully we’ve addressed all the feedback.

Kind regards

Lee Peters

1. **|** P a g e
