# Supplementary material for: What is the scope of teaching and training of undergraduate students and trainees in point of care testing in United Kingdom universities and hospital laboratories?
Source: PLoS One. 2022 Aug 1;17(8):e0268506. doi: 10.1371/journal.pone.0268506 (PMC9342762; doi:10.1371/journal.pone.0268506)
Supplement: Attachment — Submitted filename: Response to reviewers.docx [file pone.0268506.s013.docx]

RE: PONE-D-21-35377R1

Dear Editor,

Many thanks to both you and the reviewers for taking the time to re-review our manuscript and providing welcome feedback and comments.

We have gone through the manuscript and made all the required typographical and grammatical changes suggested by reviewers.

Once again, thank you for taking the time for reviewing our manuscript and hopefully we’ve addressed all the feedback.

Kind regards

Lee Peters
